# Supplementary material for: Microtubule-Dependent Mitochondria Alignment Regulates Calcium Release in Response to Nanomechanical Stimulus in Heart Myocytes
Source: Cell Rep. 2015 Dec 24;14(1):140–51. doi: 10.1016/j.celrep.2015.12.014 (PMC4983655; doi:10.1016/j.celrep.2015.12.014)
Supplement: Document S1. Supplemental Experimental Procedures, Figures S1–S7, and Tables S1 and S2 [file mmc1.pdf]

Cell Reports

Supplemental Information

Microtubule-Dependent Mitochondria Alignment Regulates Calcium Release in Response to Nanomechanical Stimulus in Heart Myocytes

Michele Miragoli, Jose L. Sanchez-Alonso, Anamika Bhargava, Peter T. Wright, Markus Sikkel, Sophie Schobesberger, Ivan Diakonov, Pavel Novak, Alessandra Castaldi, Paola Cattaneo, Alexander R. Lyon, Max J. Lab, and Julia Gorelik

Supplemental Information

Supplemental figures.

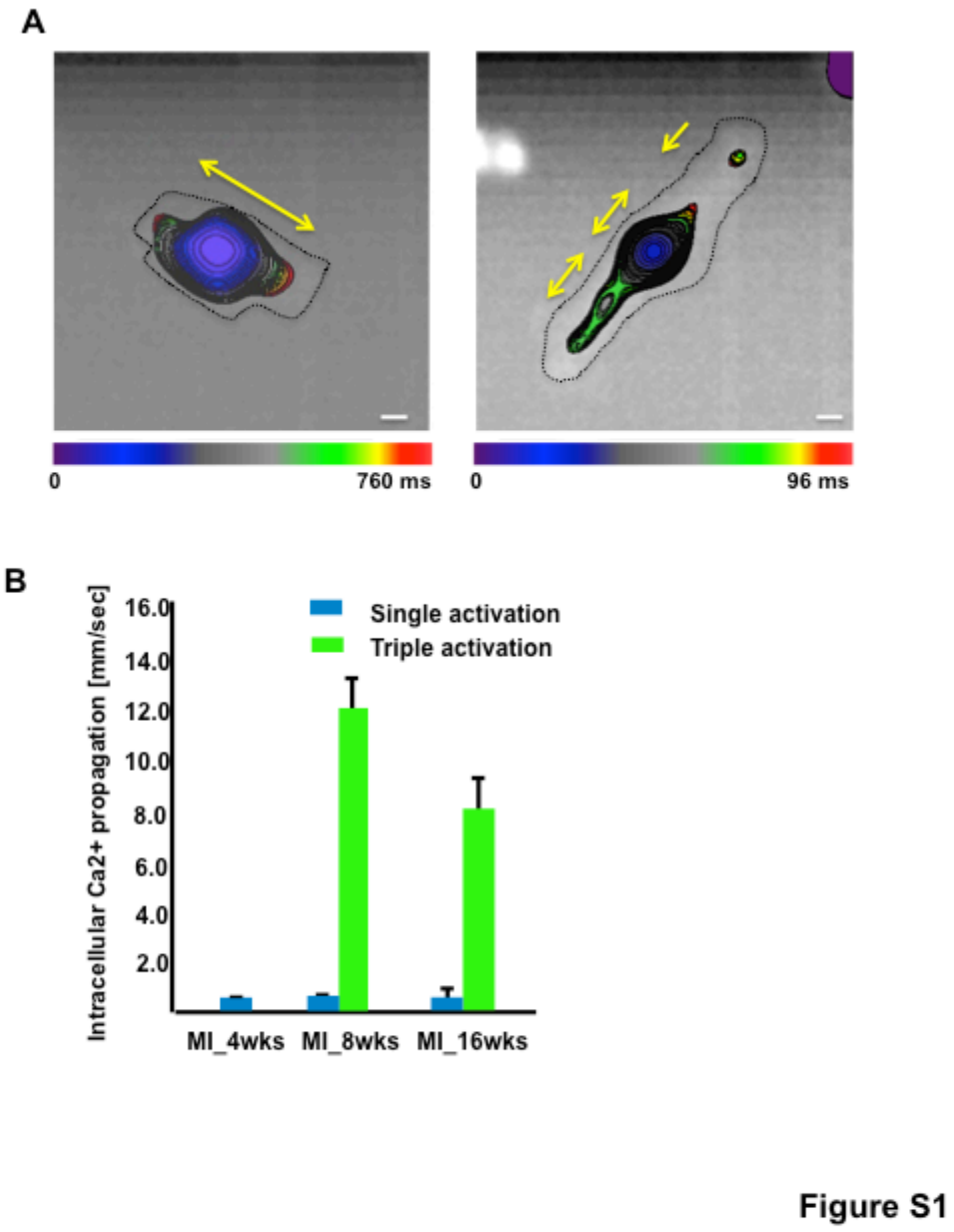

Figure S1, related to Figure 3

Propagation of MiCa$_i$ often evokes initiation of secondary calcium waves at the cell edges. A. color-coded time-lapse map of MiCa$_i$ with either single (left) or triple (right) initiation. Bar= 10 μ **B**. Intracellular calcium propagation velocity at different times following MI.

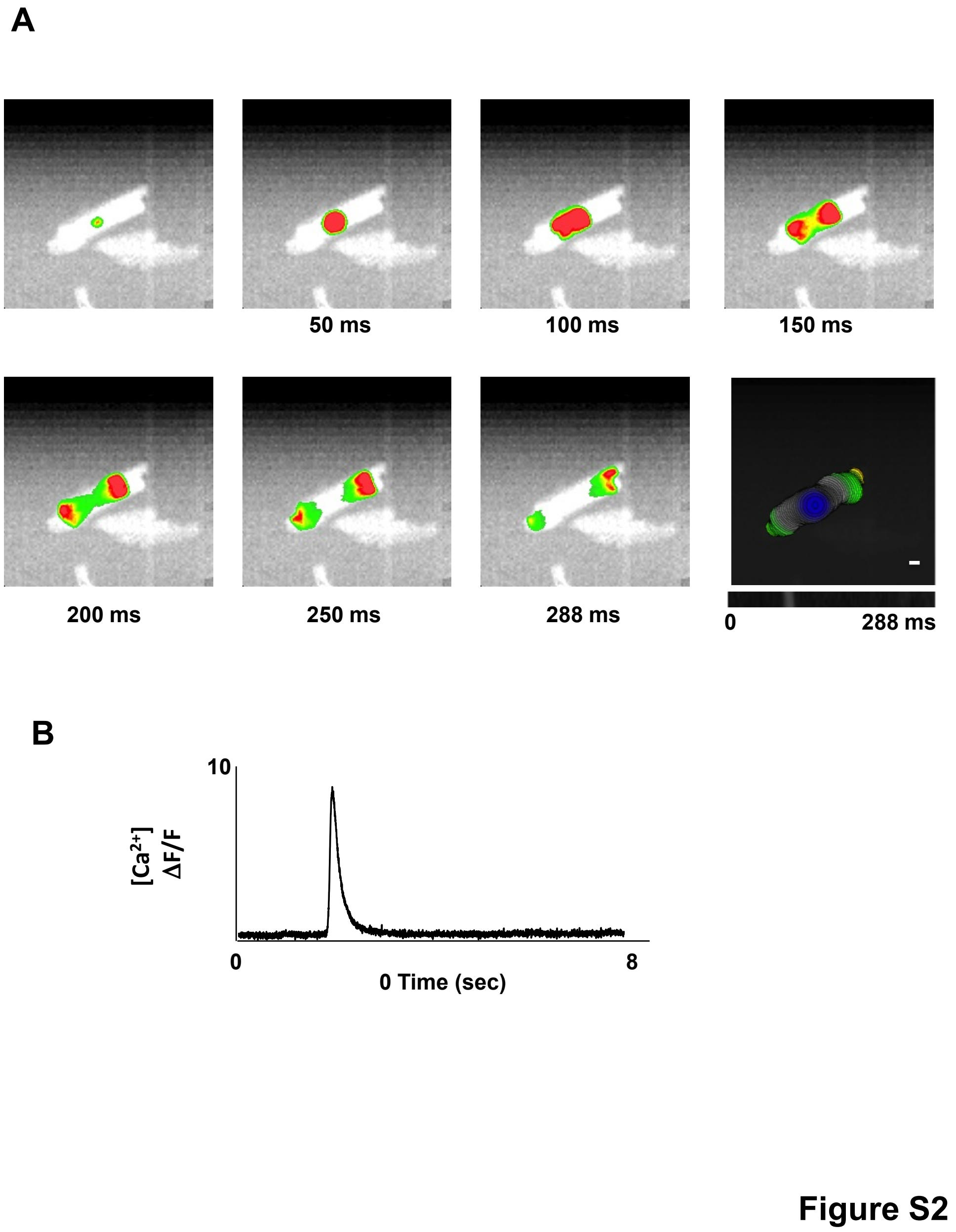

Figure S2, related to Figure 3

Inhibition of mechanosensitive ion channels does not stop MiCa$_i$ initiation. The cardiomyocytes were exposed to 30 µmol/L gadolinium. **A**. Representative frames (time interval 50 ms) of MiCa$_i$ initiated at the pressure site (upper left) and propagated as ripple effect toward the cell edges; bottom right: isochronic color-coded map). **B**. MiCa$_i$ trace for the cardiomyocyte exposed to gadolinium shown in A. Scale bar 10 µm, n=6.

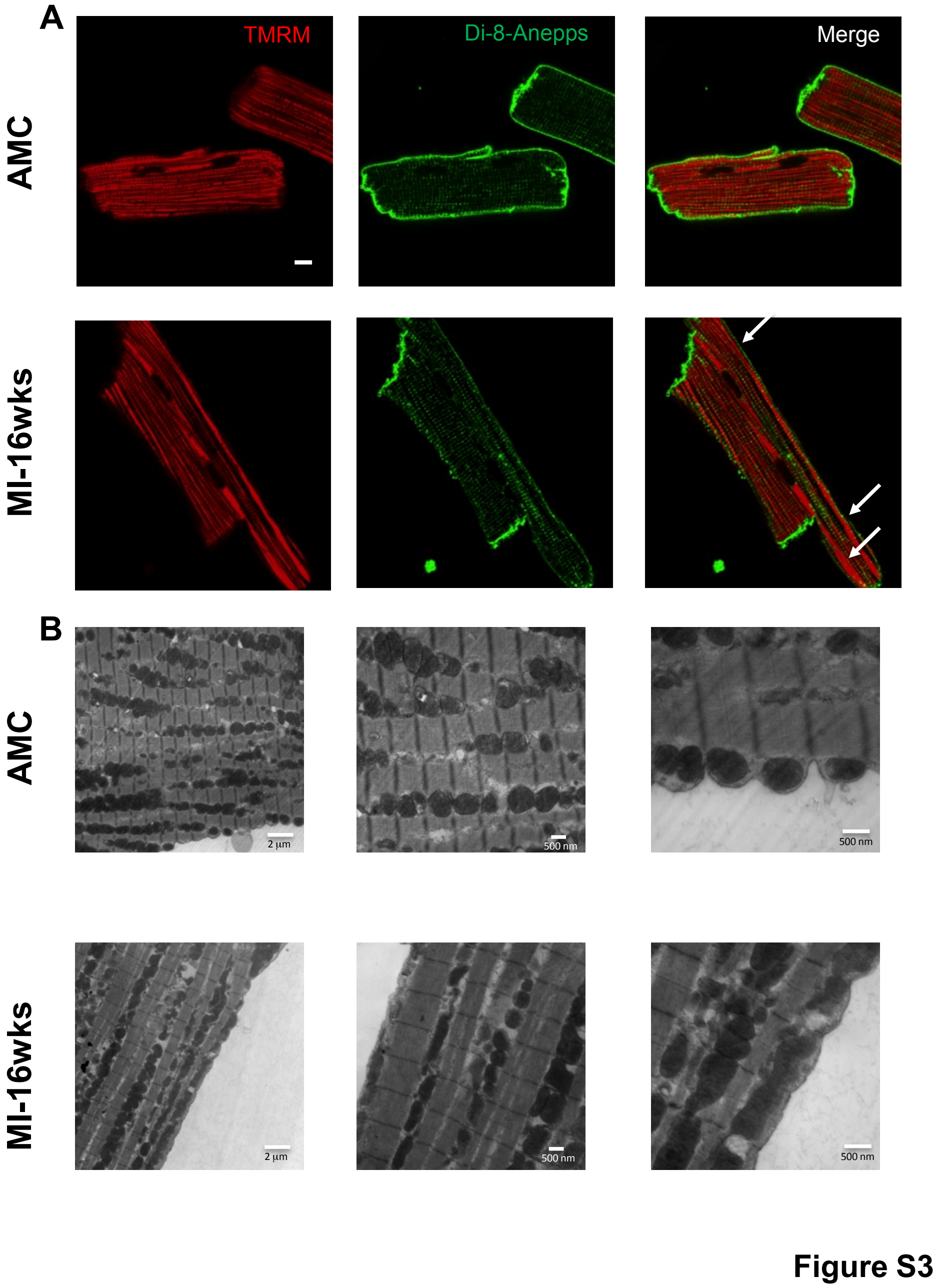

Figure S3, related to Figure 4

Remodelling of mitochondria and T-tubule network following MI. A. Mitochondria are stained with TMRM; T-tubules are stained with Di-8-ANEPPS in AMC myocytes (top row) and failing cells (bottom row). Bar= 10 µm. Arrows indicate mitochondria enlargement and relocalization. **B**. Mitochondria organization displayed in TEM images for control caridoymocytes (AMC, top) and failing cardiomyocytes (HF, bottom) showing mitochondrial enlargement at different magnification (from left to right 5000X, 10000X, 20000X).

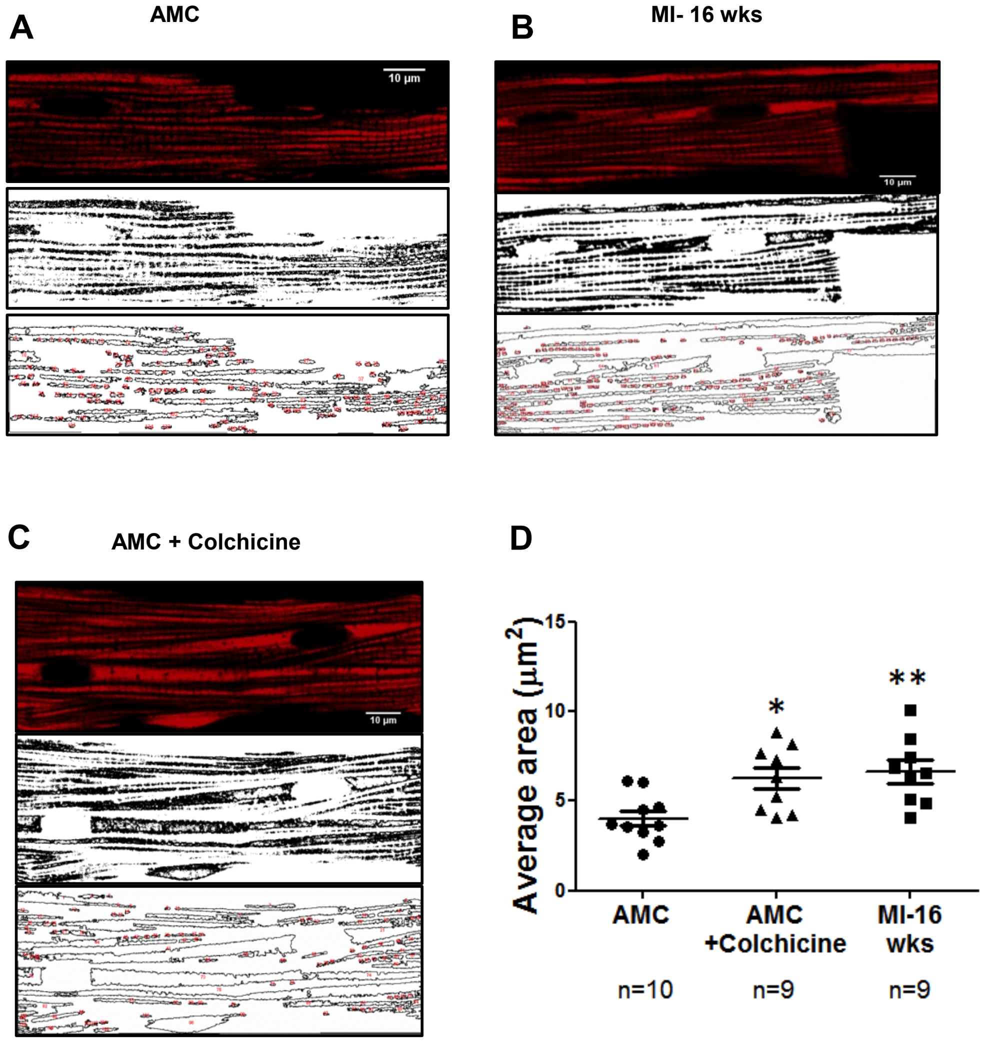

Figure S4

Figure S4, related to Figure 4

Estimation of mitochondrial elongation. A. Top: confocal image of a normal cardiomyocyte with mitochondria labeled (TMRM). Middle: the same image binarized. Bottom: regions of interest automatically selected for area calculations by a plug-in in ImageJ software. **B**. Same as A for a control cardiomyocyte exposed to colchicine. **C**. Same as A for a heart failure cardiomyocyte. **D**. Analysis of mitochondrial area in the three conditions: control, control+colchicine and heart failure. n>9. P<0.05.

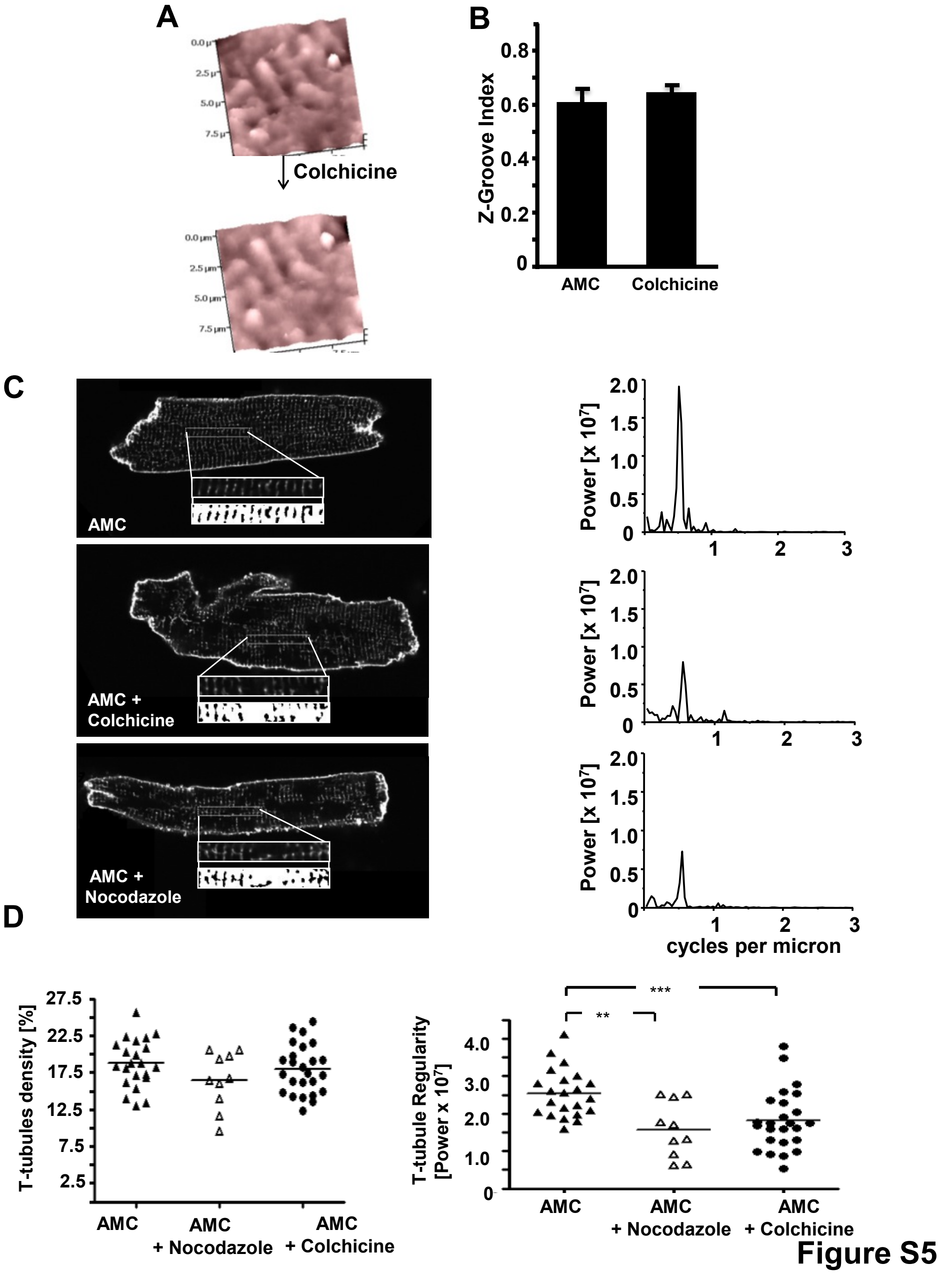

Figure S5, related to Figure 5

Effect of Colchicine on T-tubules and Z-grooves A. Topography of an AMC cardiomyocyte after treatment with colchicine (10µmol/l). **B.** Z-groove index after colchicine treatment does not change; **C.** Left: T-tubule staining with Di-8-ANEPPS after colchicine or nocodazole treatment. Insets: binarised images of selected areas on which regularity calculations have been made; Right: Power of regularity of intensity peaks corresponding to the images on the left (fast Fourier Transform analysis); **D.** T-tubule density (left panel) and regularity (right panel) after colchicine or nocodazole treatment.

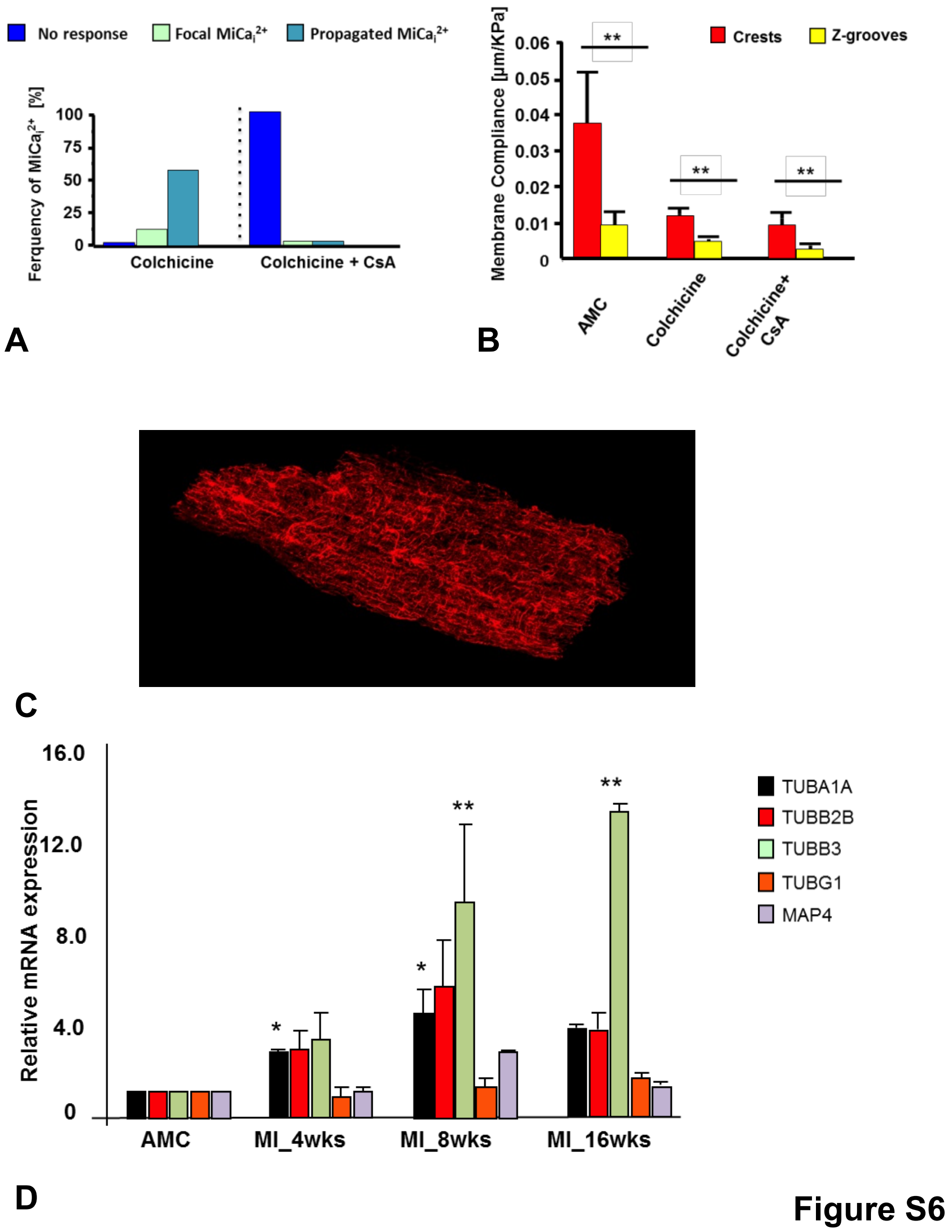

Figure S6

Figure S6, related to Figure 3 and 5

Cyclosporine A abolishes $MiCa_i$ after colchicine treatment but does not affect membrane compliance. A. Occurrence of $MiCa_i$ events in control cells subjected to colchicine and colchicine plus CsA. **B**. Membrane compliance of Z-grooves and crests in control cells, control cells subjected to colchicine and to colchicine plus CsA.

Changes in tubulin expression in HF. C. Representative image of a cardiomyocyte at 16weeks post-MI stained for β-tubulin. Scale bar is 10 µm. **D**. Relative mRNA expression for various microtubule proteins in: age matched control AMC, and different times following MI. (n=3 x technical triplicate normalized against Cyclophillin).

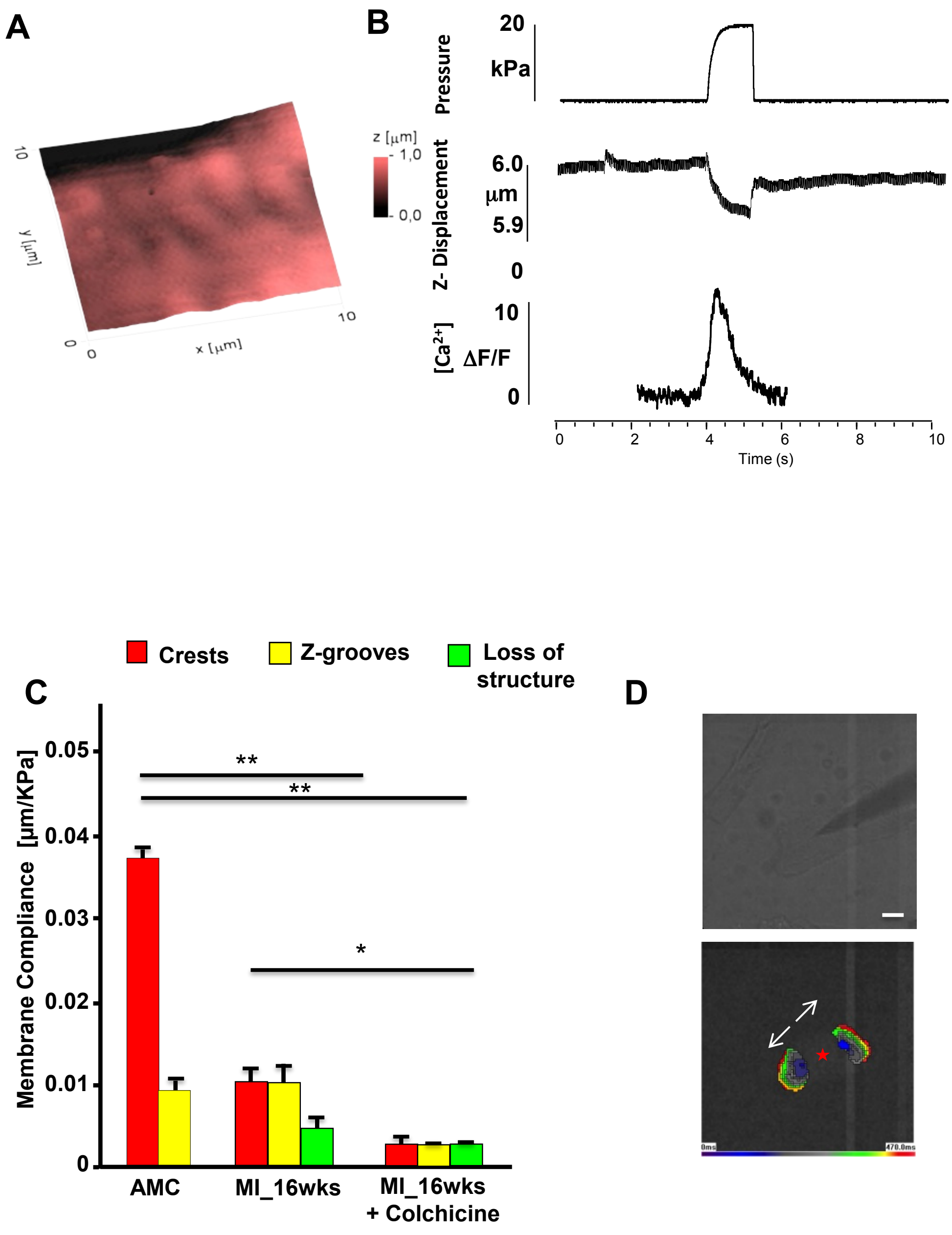

Figure S7

Figure S7, related to Figure 5

Effect of microtubular network derangement in failing cardiomyocyte. A. Representative SICM topographical image of a HF cell after colchicine treatment; **B**. Traces of mechanical application (top) membrane indentation (middle) and $MiCa_i$ transient (bottom) in failing cardiomyocytes subjected to colchicine. **C**. Membrane compliance measurement in control cardiomyocytes (AMC), failing cardiomyocytes and failing cardiomyocytes subjected to colchicine. **D**. Top: nanopipette positioned on top of a caridoymocyte for pressure application and (bottom) isochronic color-coded map of a subsequent $MiCa_i$ event.

Weeks Post MI	n	HW (mg) / TL (mm) [mean (SEM)]	n	LVEF (%)
Control	10	36.4 (2.07)	9	78.5 (3.21)
4	16	41.0 (1.10) *	5	53.5 (6.96)**
8	9	40.1 (1.43)	8	36.76 (5.90)***
16	16	46.5 (1.89) **	9	29.4 (2.02)***

Table S1

Table S1, related to Figure 2.

Validation of the heart failure model by generating myocardial infarction with coronary artery occlusion. Heart weight/Tibia length ratio (HW/TL) and left ventricular ejection fraction (LVEF) in rats measured at 4, 8 and 16 weeks after coronary ligation. ** $P<0.05$ and *** $P<0.01$

Rat Cell Type	$MiCa_i$	Surface structure	Membrane Compliance in Crest / Groove / Unstriated regions (μm/kPa)	Z-Groove Ratio, % change from AMC cells
**AMC**	Focal at Z-Grooves	Regular	0.037 / 0.009 / -	100
**AMC + Colchicine**	Focal at Z-Grooves<br>Propagated at crests	Regular	0.01. / 0.005 / -	Unchanged
**AMC + Colchicine + CCCP**	None	Regular	0.014 / 0.006 / -	Unchanged
**MI-4 wks**	Focal at Z-Grooves;<br>Propagated with single initiation	Loss of some structures	0.009 / 0.006 / 0.003	-6.8
**MI-8 wks**	Propagated with triple initiation	Non-striated	0.008 / 0.015 / 0.010	-24.7
**MI-16 wks**	Propagated with triple initiation	Non-striated	0.010 / 0.010 / 0.013	-29.8
**MI-16 wks + Colchicine**	Propagated with triple initiation	Non-striated	0.003 / 0.002 / 0.003	-28.8
**MI-16 wks + Gd^{3+}**	Propagated with single initiation	Non-striated	0.015 / 0.010 / 0.011	-27.9
**MI-16 wks + CCCP**	none	Non-striated	0.008 / 0.006 / 0.001	-30.3

Table S2, related to Figure 3.**Summary of different conditions where we observed $MiCa_i$**

Supplemental Movie Legend

Movie S1. Pattern of $MiCa_i$ propagation in MI _16wks cardiomyocytes: single initiation

Movie S2. Pattern of $MiCa_i$ propagation in MI _16wks cardiomyocytes: multiple initiations.

Movie S3. Inhibition of mechanosensitive ion channels allows only single initiation of $MiCa_i$.

Supplemental Experimental Procedure

In-vivo cardiac function

Cardiac function was assessed via biometrics and echocardiography. Heart weight corrected to tibia length provided a measure of hypertrophy. Echocardiography was performed under general anaesthesia (2% isoflurane) immediately prior despatch to give a measure of in-vivo cardiac function. The imaging was performed in M-Mode in the parasternal long axis view (Table S1, Vevo 770 system). After 4, 8 or 16 weeks following coronary ligation, rats were despatched by cervical dislocation after brief exposure to 5% isoflurane until the righting reflex was lost. We perfused the left ventricle (LV) via the Langendorff perfusion apparatus[1]. Cardiomyocytes were enzymatically isolated from the LV.

Topographical images

High-resolution cell membrane topography was achieved using high-resistance nanopipette (100MΩ). Surface topographical images (10x10 μm, 512x512 pixels) of the cardiomyocytes were acquired by the SICM at 25°C, pH=7.4. In order to avoid contraction and the same preparation was superfused with HBSS, pH=7.4 at 36 °C afterwards. After acquiring the topography image, the pipette was moved to a selected location on the cell surface 200 nm above a cell crest or groove by a controlled movement of the piezo drive.

Transmission electron microscopy

Isolated cells were fixed in 2.5% glutaraldehyde in cacodylate buffer, cell pellets were embedded in 2% agarose and re-fixed in 2.5% glutaraldehyde, post-fixed in 1% osmium trioxide and embedded in Araldite following standard protocol. Ultra-thin sections were mounted on grids and stained for 7 minutes with 2% uranyl acetate in methanol, washed again in methanol, and stained for 5 minutes with 1% led citrate in water and washed in water afterwards. The sections were observed with transmission electron microscope.

Immunostaining

Adult cardiomyocytes were stained for β-tubulin (Monoclonal Anti-β -Tubulin; t5201; Sigma-Aldrich; Alexa 546 anti-mouse immunoglobulin; Abcam) by a standard indirect immunofluorescence protocol. Cells on coverslips were fixed in 4% paraformaldehyde in PBS for 10 minutes, permeabilized in 1% triton X100 in PBS for 20 minutes; washed in PBS twice, incubated in blocking solution (5% BSA, 20% Newborn calf serum, 0.05% Tween 20 in PBS) for 30 minutes They were then incubated with first antibody (diluted 1:100) in blocking solution overnight, washed in PBS twice, incubated with secondary antibody (diluted 1:100) in blocking solution for 30 minutes. They were subsequently mounted in Vectashield mounting medium (Vector Labs), sealed with nail varnish, and observed with confocal microscope (Zeiss LSM-780).

Supplemental reference.

1. Sato M, O'Gara P, Harding SE, Fuller SJ. Enhancement of adenoviral gene transfer to adult rat cardiomyocytes in vivo by immobilization and ultrasound treatment of the heart. *Gene Ther*. 2005;12:936-941
