# Supplementary figures and images for: Microtubule-Dependent Mitochondria Alignment Regulates Calcium Release in Response to Nanomechanical Stimulus in Heart Myocytes
Source: Cell Rep. 2015 Dec 24;14(1):140–51. doi: 10.1016/j.celrep.2015.12.014 (PMC4983655; doi:10.1016/j.celrep.2015.12.014)

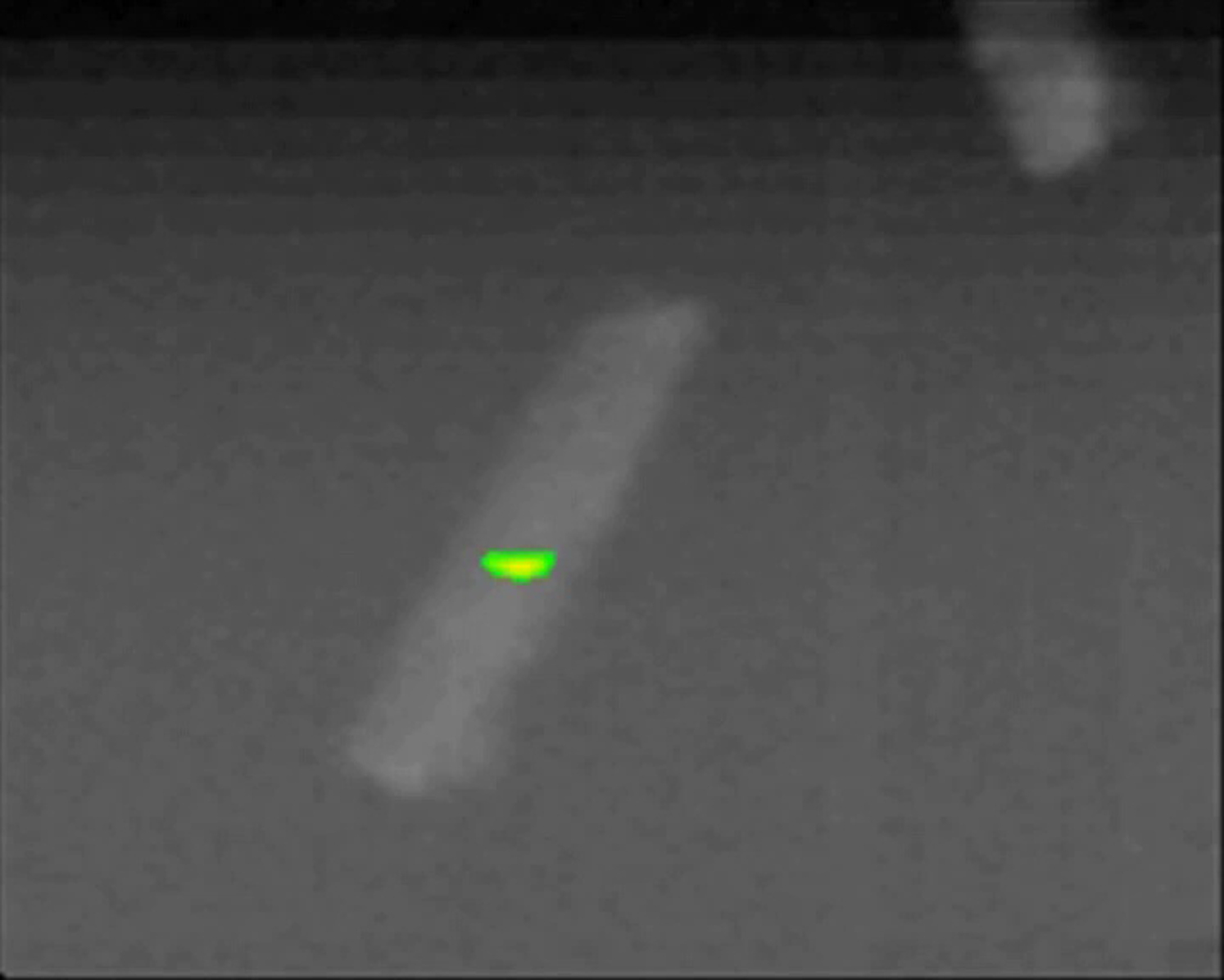

Supplement: Movie S1. Pattern of MiCai Propagation in MI _16wks Cardiomyocytes: Single Initiation [file mmc2.jpg]

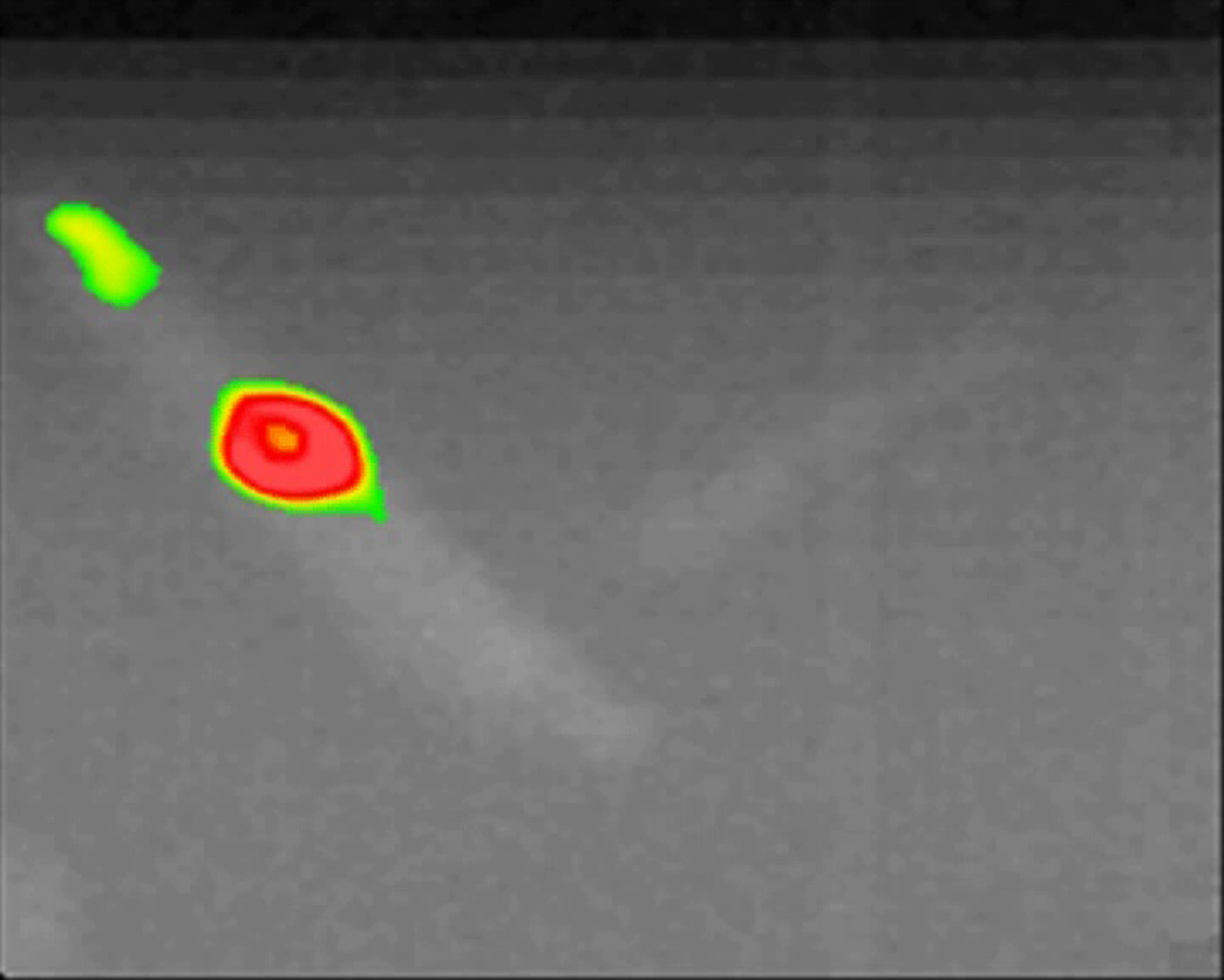

Supplement: Movie S2. Pattern of MiCai Propagation in MI _16wks Cardiomyocytes: Multiple Initiations [file mmc3.jpg]

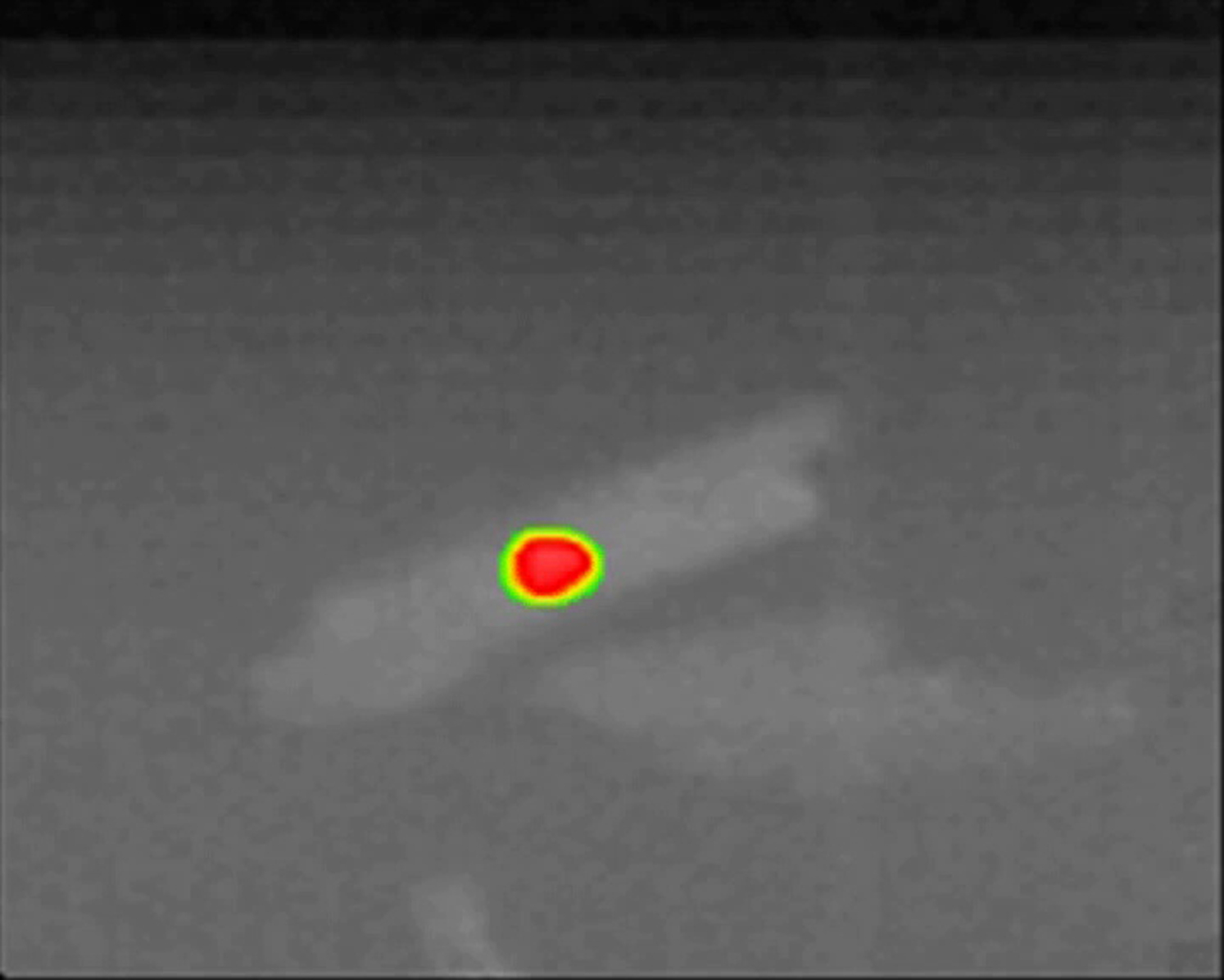

Supplement: Movie S3. Inhibition of Mechanosensitive Ion Channels Allows Only Single Initiation of MiCai [file mmc4.jpg]
