# Supplementary material for: Microtubule-Dependent Mitochondria Alignment Regulates Calcium Release in Response to Nanomechanical Stimulus in Heart Myocytes
Source: Cell Rep. 2015 Dec 24;14(1):140–51. doi: 10.1016/j.celrep.2015.12.014 (PMC4983655; doi:10.1016/j.celrep.2015.12.014)
Supplement: Document S2. Article plus Supplemental Information [file mmc5.pdf]

Cell Reports

Article

Microtubule-Dependent Mitochondria Alignment Regulates Calcium Release in Response to Nanomechanical Stimulus in Heart Myocytes

Graphical Abstract

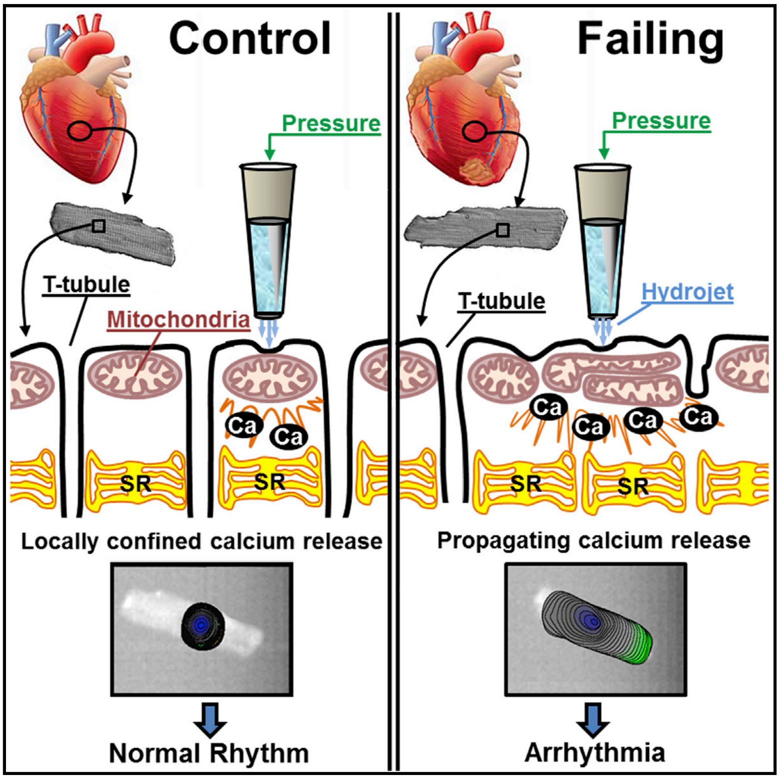

Authors

Michele Miragoli, Jose L. Sanchez-Alonso, Anamika Bhargava, ..., Alexander R. Lyon, Max J. Lab, Julia Gorelik

Correspondence

michele.miragoli@humanitasresearch.it (M.M.), m.lab@imperial.ac.uk (M.J.L.), j.gorelik@imperial.ac.uk (J.G.)

In Brief

Miragoli et al. show that failing heart cells have altered sensitivity to nanomechanical stimuli mediated by changes in the alignment of microtubules. The microtubule network disorganization leads to displacement of mitochondria and alterations in calcium release.

Highlights

- Nanomechanical pressure application changes mechanosensitivity in failing heart cells
- Microtubular network disorganization mediates the change in mechanosensitivity
- Mitochondria are displaced from their original location and trigger calcium release
- Uncoupling the mitochondrial proton gradient completely abolishes the phenomena

Miragoli et al., 2016, Cell Reports *14*, 140–151
January 5, 2016 ©2016 The Authors
http://dx.doi.org/10.1016/j.celrep.2015.12.014

OPEN ACCESS
CellPress

Cell Reports
Article

Microtubule-Dependent Mitochondria Alignment Regulates Calcium Release in Response to Nanomechanical Stimulus in Heart Myocytes

Michele Miragoli,[1,2,3,*] Jose L. Sanchez-Alonso,[1] Anamika Bhargava,[1,6] Peter T. Wright,[1] Markus Sikkel,[1] Sophie Schobesberger,[1] Ivan Diakonov,[1] Pavel Novak,[1,5] Alessandra Castaldi,[2] Paola Cattaneo,[2] Alexander R. Lyon,[1,4] Max J. Lab,[1,*] and Julia Gorelik[1,*]
[1]National Heart and Lung Institute, Imperial College London, 4th floor, Imperial Centre for Translational and Experimental Medicine, Hammersmith Campus Du Cane Road, London W12 0NN, UK
[2]Humanitas Clinical and Research Center, via Manzoni 56, Rozzano, 20090 Milan, Italy
[3]Center of Excellence for Toxicological Research, INAIL exISPESL, University of Parma, via Gramsci 14, 43126 Parma, Italy
[4]NIHR Cardiovascular Biomedical Research Unit, Royal Brompton Hospital, London SW36NP, UK
[5]School of Engineering and Materials Science, Queen Mary, University of London, Mile End Road, London E1 4NS, UK
[6]Department of Biotechnology, Indian Institute of Technology Hyderabad, Ordnance Factory Estate, Yeddumailaram, 502205 Telangana, India
*Correspondence: michele.miragoli@humanitasresearch.it (M.M.), m.lab@imperial.ac.uk (M.J.L.), j.gorelik@imperial.ac.uk (J.G.)
http://dx.doi.org/10.1016/j.celrep.2015.12.014
This is an open access article under the CC BY-NC-ND license (http://creativecommons.org/licenses/by-nc-nd/4.0/).

SUMMARY

Arrhythmogenesis during heart failure is a major clinical problem. Regional electrical gradients produce arrhythmias, and cellular ionic transmembrane gradients are its originators. We investigated whether the nanoscale mechanosensitive properties of cardiomyocytes from failing hearts have a bearing upon the initiation of abnormal electrical activity. Hydrojets through a nanopipette indent specific locations on the sarcolemma and initiate intracellular calcium release in both healthy and heart failure cardiomyocytes, as well as in human failing cardiomyocytes. In healthy cells, calcium is locally confined, whereas in failing cardiomyocytes, calcium propagates. Heart failure progressively stiffens the membrane and displaces sub-sarcolemmal mitochondria. Colchicine in healthy cells mimics the failing condition by stiffening the cells, disrupting microtubules, shifting mitochondria, and causing calcium release. Uncoupling the mitochondrial proton gradient abolished calcium initiation in both failing and colchicine-treated cells. We propose the disruption of microtubule-dependent mitochondrial mechanosensor microdomains as a mechanism for abnormal calcium release in failing heart.

INTRODUCTION

Pump failure and sudden cardiac death remain a major clinical problem despite conventional therapies. Altered mechanosensitivity initiates electrical instability and arrhythmia in heart failure (Kiseleva et al., 2000). Whereas pro-arrhythmic mechanoelectric transduction has been extensively investigated in intact hearts in situ, isolated hearts, and in isolated cellular preparations, the initial subcellular mechanisms required for signal transduction and its initiation remain elusive (Lammerding et al., 2004). Recent attention has focused upon different sarcomeric components (Kim et al., 1999), and in addition to force generation, several sarcomeric proteins were found to provide mechanosensing and/or signaling functions (Borg et al., 2000; Knöll et al., 2002). Mutations in these sarcomeric or Z-disk complex proteins cause abnormal intracellular Ca^{2+} responses (Knöll et al., 2002).

During heart failure, the cytoskeletal scaffold remodels, and this may also disturb the normal regulation of mechanosensation (Janmey and Miller, 2011). Loss of appropriate mechanical feedback control may contribute to the development of heart failure. The structural remodeling that occurs during heart failure involves the cell membrane (loss of T-tubules; Lyon et al., 2009), intercalated disks (Ferreira-Cornwell et al., 2002), and sub-membrane microdomains involving ryanodine receptors (RyRs) and the sarcoplasmic reticulum (Dobrev and Wehrens, 2014). Importantly, mitochondria change their subcellular location (Piquereau et al., 2013; Rosca et al., 2013) and the inter-fibrillar mitochondria alignment is altered early following myocardial infarction (Dague et al., 2014). Regular alignment of mitochondria and the dyad plays a pivotal role in the homeostasis of excitation-contraction coupling (Chen et al., 2012; Kohlhaas and Maack, 2013; Lu et al., 2013) and intracellular calcium handling (Belmonte and Morad, 2008b). However, little is known about the possible role of mitochondria remodeling in mechanoelectric transduction-induced arrhythmia. This reflects the inability of many conventional technologies to selectively and mechanically activate or investigate mitochondrial involvement within a single sarcolemmal microdomain. Here, we have employed scanning ion conductance microscopy (SICM) and surface

CrossMark

OPEN
ACCESS
CellPress

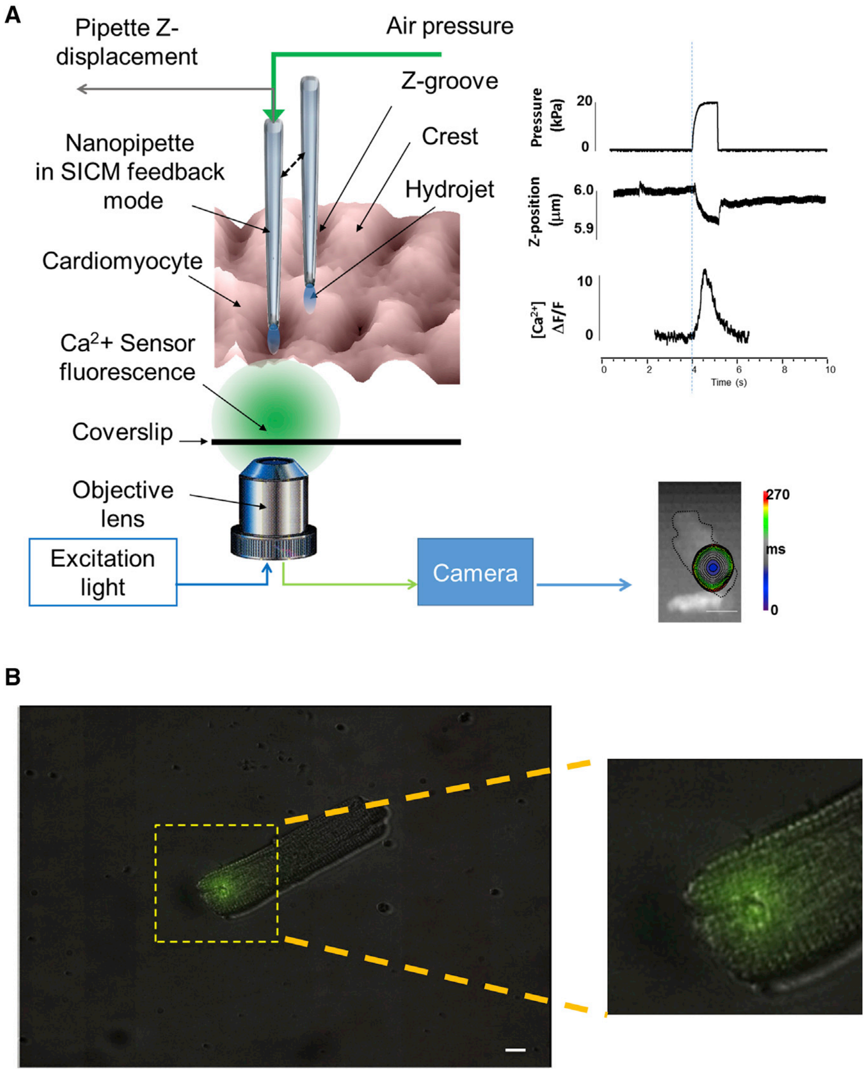

Figure 1. Schematic Representation of the Experimental Protocol

(A) Cells were loaded with 5 μmol/l of Fluo-4 AM, and a 10 × 10 μm of cell surface was scanned with the SICM. The nanopipette was positioned above a crest or a groove as identified on the scan and, while keeping the distance constant at 200 nm, positive air pressure was applied to the auxiliary port of the pipette holder, generating a hydrojet pressure. A protocol written on Clampfit 10.0 (Molecular Devices) synchronized the light shutter for optical acquisition (8 s in total at 1–5 KHz temporal acquisition) and the pump for pressure application (ramp duration 2 s at 20 kPa). Fluo-4 fluorescence emission was recorded (represented as a color-coded time-lapse map) together with the Z-piezo displacement (corresponding to membrane indentation) and the mechanically induced calcium initiation and propagation. Typical readings of pressure, Z-piezo displacement, and calcium transient are represented on the right.

(B) Perturbed area following 20 kPa ramp hydrojet pressure (2 s) in an isolated cardiomyocyte. Pipette solution was filled with 1 μM Lucifer yellow, resulting in ~0.125 μm^2 area (green spot), enlarged in the inset.

The scale bar represents 10 μm in (A) and 500 nm in the inset.

confocal SICM to resolve cellular topography and mitochondria localization.

We applied pressure via the SICM nanopipette with nanometer precision to investigate the subcellular mechanisms underlying mechanically induced calcium release in heart failure (Figure 1). Hydrojets targeting structurally regular Z-grooves in healthy cardiomyocytes caused mechanically induced intracellular calcium release ($MiCa_i$) events, which were spatially confined. By contrast, $MiCa_i$ propagated throughout the cell in failing cardiomyocytes with irregular Z-grooves. We found that the likelihood of producing propagating $MiCa_i$ correlated with the degree of mitochondrial derangement within the dyad as well as with a decrease in membrane compliance at the point of applied mechanical force. Having excluded mechanosensitive ion channels and actin filaments as the mechanosensing substrates, we observed that propagating $MiCa_i$ could be simulated by disrupting microtubules in healthy cardiomyocytes, which are responsible for maintaining sub-sarcolemmal mitochondrial positions (Saetersdal et al., 1990). Uncoupling the mitochondrial proton gradient abolishes $MiCa_i$ propagation. It appears that microtubules associated with mitochondria may represent a signaling microdomain that responds to mechanical stimulation of the sarcolemma. This study suggests that microtubular and mitochondrial derangement play pivotal roles in the initiation of abnormal calcium release during progression toward heart failure and provides an additional mechanism for non-action potential-mediated intracellular calcium release, which could lead to triggered activity and arrhythmias.

RESULTS

Structural and Mechanical Properties of Failing Cardiomyocytes

We scanned the structural features of the sarcolemma of normal and failing cardiomyocytes (mainly from compensatory hypertrophy, derived from zones remote from the scar) using SICM at different time points following myocardial infarction (MI). Then, we positioned the pipette at 200 nm over a pre-selected site on the sarcolemma (either a crest or a Z-groove or an area without structure) chosen on a previously acquired topography image (Figure 1A). Subsequently, we applied a localized 20-kPa hydrojet for 2 s. The area indented by the hydrojet (Figure 1B) is in the range of 0.125 μm^2.

We studied rat cardiomyocytes following MI, progressing toward heart failure. They developed heart failure in our model at

OPEN
ACCESS
CellPress

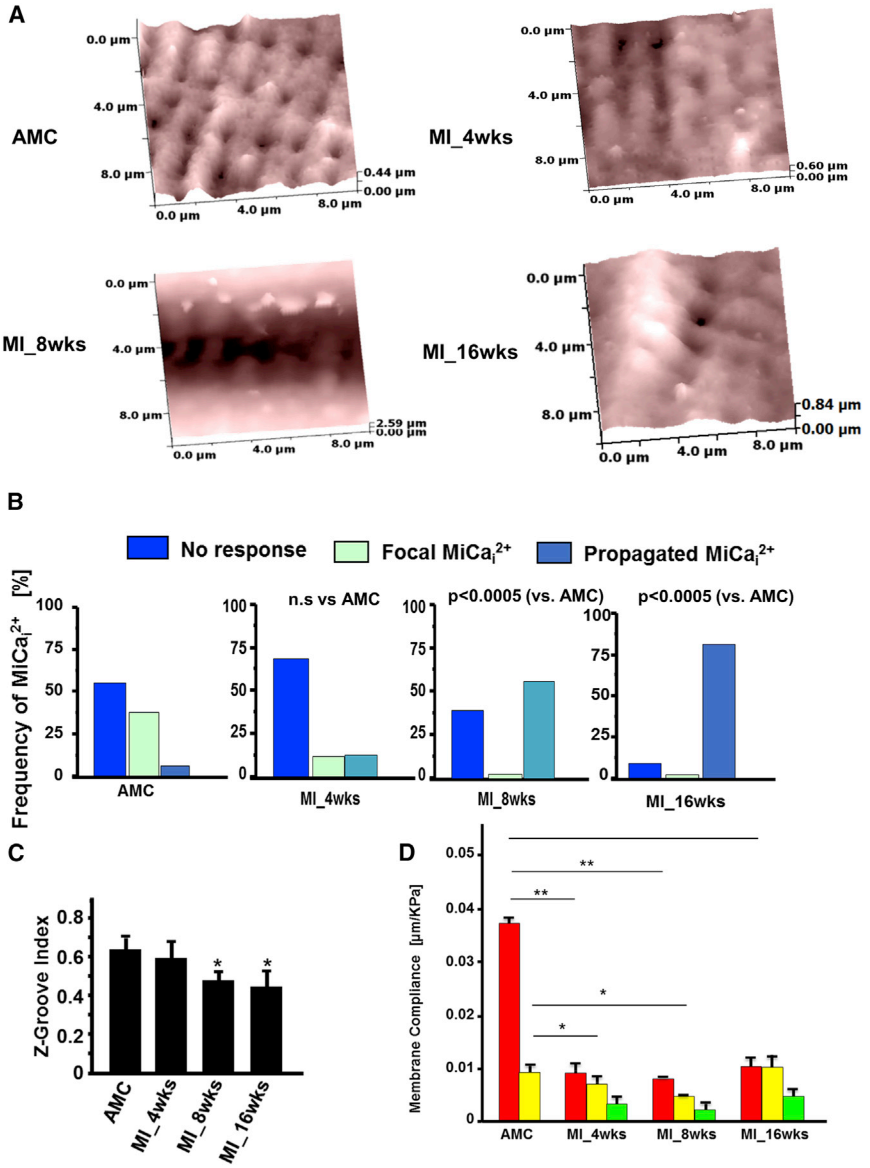

Figure 2. MiCa$_i$ Propagation Changes from Local to General during Progression to Heart Failure

(A) (Upper left) Surface topography of an AMC cardiomyocyte (10 × 10 μm). (Upper right) Surface topography of a heart failure cardiomyocyte (10 × 10 μm) at 4 weeks post-myocardial infarction (MI_4wks) is shown. (Lower left) MI_8wks surface topography is shown. (Lower right) MI_16wks surface topography is shown.

(B) Frequency of MiCai propagation during progression to heart failure at AMC, 4–8, and 16 weeks post-MI, respectively.

(C) Z-groove index calculated for AMC cells and heart failure cells at 4, 8, and 16 weeks post-MI (n = 6 each; mean ± SEM; *p < 0.0005).

(D) Membrane compliance calculated after 20 kPa hydrojet square pulse pressure applied for 2 s at crests, Z-grooves, or un-striated parts of the cells. Pipette-tip diameter 200 nm; n: approximately 20 cells each group; n = 71 in total; mean ± SEM; *p < 0.05; **p < 0.001.

16 weeks with clear evidence of hypertrophy and left ventricular failure (Table S1). We first obtained a 10 × 10 μm SICM topographical image of a normal or a failing cardiomyocyte (Figure 2A). These scanned topographical images were used to quantify disruption of surface structural regularity; the images showed that cells progressively change their sarcolemmal regularity (Figure 2A). We measured the Z-groove index, as previously reported (Lyon et al., 2009, 2012), which reduced significantly from 0.62 ± 0.16 in control cardiomyocytes to 0.44 ± 0.19 in cells 16 weeks post-MI ($p < 0.05$; Figure 2B). Membrane organization is substantially altered following MI, including the disappearance of crests and grooves.

To study the mechanical properties of the cell surface microdomains, we then applied pressurized hydrojet ramps within the range 0–40 kPa (typically 20 kPa) for 2 s either over a smooth or grooved area of the cell and recorded pipette vertical displacement. As the pipette, driven by the SICM's feedback control mechanism, follows the cell surface under the pipette, we essentially recorded membrane displacement (Z) as a function of applied pressure. In normal cells, the areas around grooves are stiffer than the crests, as less pipette displacement was observed for the same pressure applied. However, failing cells have uniformly stiff membranes, regardless of the area (Figure 2D).

During progressive heart failure, we found that, at 4 weeks after MI, the surface regularity begins to change, but not significantly (Figures 2A and 2C). However, membrane compliance has already reduced significantly in all areas of the sarcolemma (Figure 2D). At 8 weeks, structure is gradually lost and the membrane is stiffer than in control cells. The membrane compliance data following hydrojet application have been previously used to calculate Young's modulus of elasticity in living cells (Sánchez et al., 2008). We found, similarly, that the modulus varies considerably across the myocyte surface (crest: 0.038 ± 0.003 μm/kPa versus groove: 0.009 ± 0.001 μm/kPa in control cardiomyocytes; Figure 2D).

MiCa$_i$ in Failing Cardiomyocytes

In healthy control cardiomyocytes, pressure applied in a Z-groove initiates a focal MiCa$_i$, which is characterized by relatively slow propagation and is spatially restricted to the pressure site (Figure 3A, left). In contrast, in failing cardiomyocytes, MiCa$_i$ initiates at the pressure site and propagates throughout the whole cell (Figure 3A, right). The MiCa$_i$ wave spreads more rapidly in failing cells (lower time to peak; Figure 3B), as control

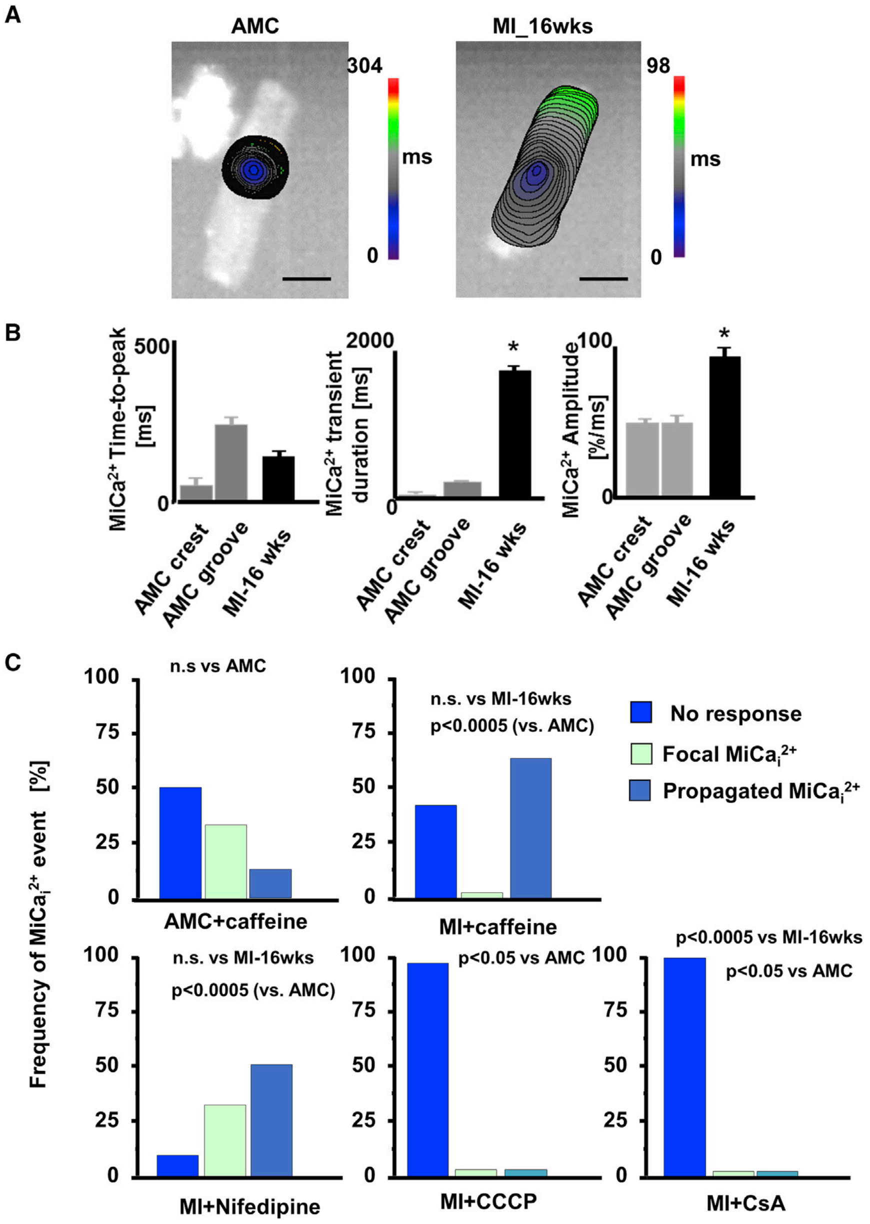

Figure 3. MiCa$_i$ Propagation Changes from Local to General during Progression to Heart Failure

(A) Color-coded propagation time maps of MiCa$_i$ in an AMC (focal propagation; left panel) and a failing cardiomyocyte 16 weeks post-MI (whole-cell propagation; right panel). The scale bar represents 10 μm.

(B) Mechanically induced calcium transient (MiCa$_i$) parameters (time-to-peak, duration, and amplitude; mean ± SEM) in AMC cells when the pressure was applied either to a crest or to a groove and in heart failure cells to unstructured areas; n = 10 each.

(C) Frequency of MiCa$_i$ in AMC cells at baseline (upper left), in the presence of caffeine (upper right), nifedipine (lower left), CCCP (lower middle), and CsA (lower right); n = 14. n.s., not significant; p = 0.0005 (MI-16wks); Fisher exact test; multiple contingency.

cardiomyocytes displayed mainly localized MiCa$_i$, with the total time to peak of 252.4 ± 11 ms, whereas failing myocytes have mainly propagated MiCa$_i$ with a time to peak of 134 ± 26 ms (Figure 3B). We studied MiCa$_i$ characteristics and kinetics. MiCa$_i$ events in failing cells have a longer duration and higher amplitude than in control cardiomyocytes (Figure 3B). As cells remodel following MI and progress toward heart failure, the probability and frequency of MiCa$_i$ propagation increases. At 4 weeks after MI, the propagated MiCa$_i$ occurs only marginally more frequently than in control cells, whereas at 8 weeks, the propagation manifests more often after a hydrojet (Figure 2B).

Generally, failing cardiomyocytes show two different patterns of MiCa$_i$ initiation and propagation (Figure S1; Movie S1). One is the appearance of a solitary "ripple" starting underneath the pressure site and slowly propagating throughout the cell (Figure S1A, left panel); this single initiation occurs at all time points at 4, 8, and 16 weeks post-MI (Figure S1B). The other initiation is more complex (Figure S1A, right panel) with the MiCa$_i$ ripple starting underneath the pressure site, but after ~1 or 2 ms, an additional remote MiCa$_i$ signal or signals (binary emergence) from the cell periphery follows the initial wave. The latter triplet Ca^{2+} wave fronts collide and propagate rapidly throughout the cell (Figure S1B; Movie S2).

MiCa$_i$ Initiation Is Independent of L-type Calcium Channels, Sarcoplasmic Reticulum, Stretch-Activated Channels, or Actin Cytoskeleton

The prime source of the initial calcium release in the cytoplasm during myocyte contraction is L-type calcium channels (LTCCs) (Santulli and Marks, 2015). To explore the involvement of LTCCs and ryanodine receptors (RyR2) in the initiation of MiCa$_i$, we varied Ca^{2+} concentration in the extracellular HBSS solution from zero, to low (0.1 μmol/l), to "physiological" (1.8 μmol/l). This doesn't influence the frequency of MiCa$_i$ (data not shown). Nifedipine also failed to stop MiCa$_i$ occurring (Figure 3C, bottom left panel). We then sought to explore the mechanosensing role of sarcoplasmic reticulum and the role of the RyR2 by analyzing the effect of caffeine on the frequency of MiCa$_i$ events. Although high-dose caffeine opens RyR2 (Dobrev and Wehrens, 2014), it doesn't alter the frequency of propagated MiCa$_i$ in both normal and failing cells (Figure 3C, top panels). We subsequently checked other potential mechanosensors that could trigger a MiCa$_i$ initiation.

OPEN ACCESS
CellPress

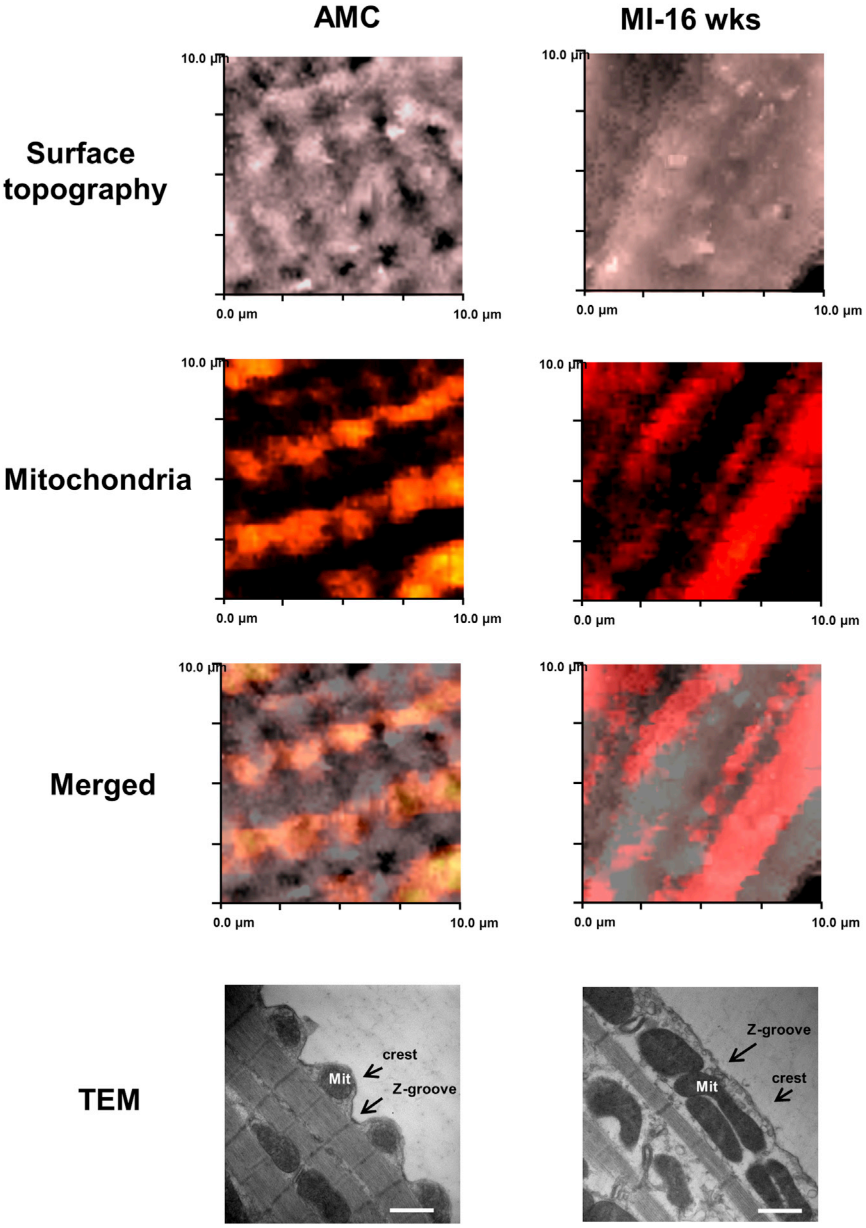

Figure 4. Myocardial-Infarction-Induced Remodeling of Dyad Microdomains Is Characterized by a Mitochondrial Shift

(Left column) Control (AMC) cells; (right column) heart-failure-derived cells (16 weeks post-MI). (Top row) SICM surface topography is shown; (next row down) TMRM-labeled mitochondria are shown; (next row down) merged images of SICM cell topography and surface confocal (10 × 10 μm) are shown; and (bottom row) representative transmission electron micrographs, illustrating the reorganization of mitochondria in heart failure, are shown.

As hydrojet indents and therefore stretches the membrane, we inhibited stretch-activated channels with either 100 μmol/l streptomycin (White, 2006) or 30 μmol/l gadolinium (Gd^{3+}) (Ermakov et al., 2010; Figure S3A; Movie S3). This failed to abolish $MiCa_i$ initiation under the pressure site, suggesting that the main local mechanosensors are not stretch-activated channels related (Figure S3B). We focused on the cytoskeleton, as many proteins at the costamere are actin-binding mechanosensing proteins (Ingber, 1997). Disrupting actin microfilaments in AMC cells with 5 μmol/l cytochalasin D, for 2 hr (Undrovinas and Maltsev, 1998), stiffened the cardiomyocyte sarcolemma and blocked contraction but did not alter $MiCa_i$ events incidence or Z-groove ratio (data not shown).

Mitochondria Re-alignment during Heart Failure Is Related to Triggering $MiCa_i$

The lack of involvement of extracellular calcium indicates the existence of an intracellular Ca^{2+} source, and having excluded the sarcoplasmic reticulum, we needed to find another source. The involvement of mitochondria in pressure-induced intracellular Ca^{2+} release has been demonstrated previously (Belmonte and Morad, 2008a), so we employed both confocal microscopy in combination with SICM (SSCM) and transmission electron microscopy (TEM) to investigate the sub-membrane interaction between dyads and mitochondria in failing cells. In normal control cardiomyocytes, active TMRM-labeled mitochondria align with crests with a periodic arrangement, which reflects regular arrangement of Z-grooves and T-tubule openings (Figures 4, particularly shown in the TEM panel, and S2A). Heart failure cells lose this regularity of mitochondria organization; it also seems that mitochondria elongate (Figure 4, right panels) and become less fragmented (Figure S2B) and the average area of mitochondria increases (Figures S6A, S6B, and S6D). Then, we inhibited the mitochondrial proton gradient and the permeability transition pore with CCCP and cyclosporinA (CsA), respectively, and, in contrast to previous pharmacological treatments described in the previous chapter, we found that this treatment abolishes the propagating $MiCa_i$ in failing cells. This indicates an active role of mitochondria in this process (Figure 3C, bottom two right panels). These observations suggest a correlation of mitochondria derangement with the occurrence of propagated $MiCa_i$ and indicate a possible active role of remodeled mitochondria microdomains in $MiCa_i$ initiation.

Microtubular Network Derangement Is Responsible for Mitochondrial Displacement

Recently, a microtubular role for $MiCa_i$ and Ca^{2+}_i spark generation has been proposed (Prosser et al., 2013; Iribe et al., 2009).

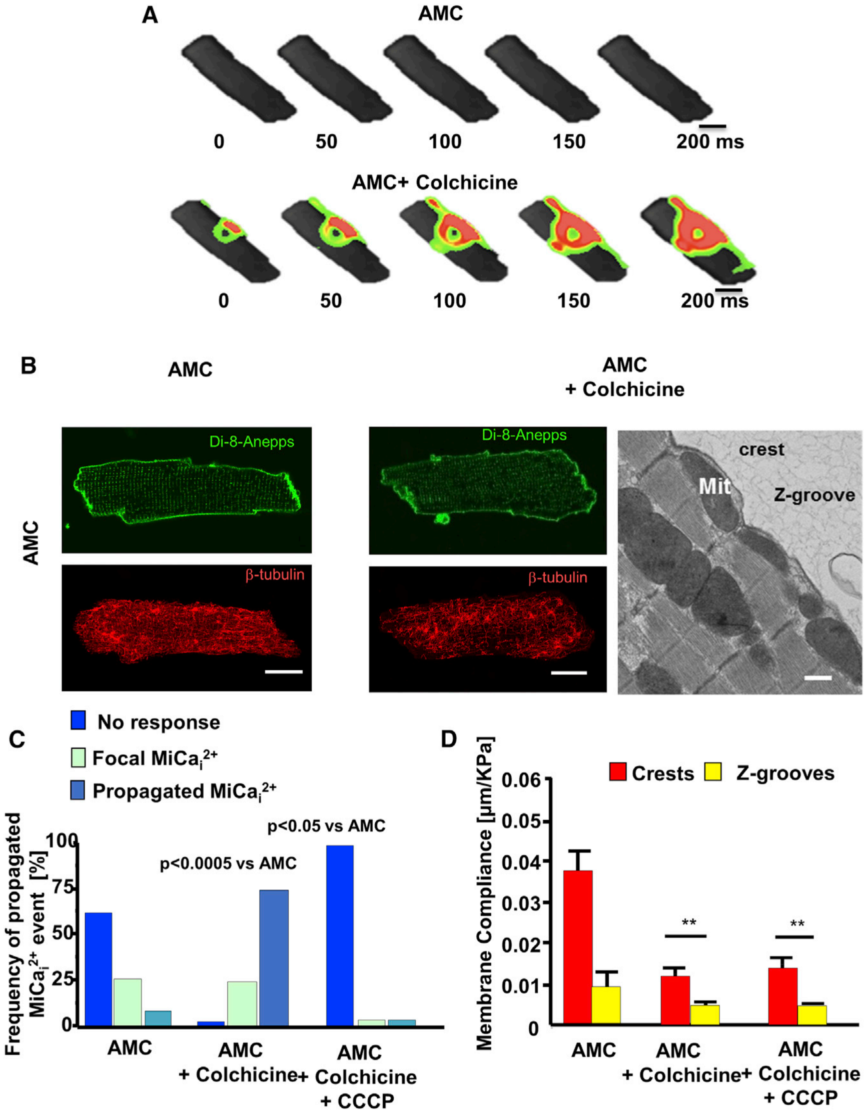

Figure 5. Disruption of Microtubules Leads to a More-Frequent MiCa$_i$

(A) Time-lapse color-coded maps of MiCa$_i$. (Top row) 20 kPa hydrojet pressure applied to the center of an AMC myocyte produces no MiCa$_i$; (bottom row) the same cell after exposure to 10 μmol/l colchicine for 1 hr at 36°C shows a propagated MiCa$_i$ after the same pressure has been applied to the same spot. The scale bar represents 10 μm.

(B) Membrane staining of T-tubules (green; Di-8-ANNEPS) and immunostaining for β-tubulin (red) in an AMC cardiomyocyte (left panels) and an AMC cardiomyocyte incubated with colchicine for 1 hr in 36°C (right panels). The scale bar represents 10 μm. (Rightmost picture) Electron micrograph shows mitochondrial movement following incubation of an AMC cell with colchicine (10 μmol/l for 1 hr). The scale bar represents 1 μm. Mit, mitochondria.

(C) Frequency of propagated MiCa$_i$ that occur in AMC cells treated with colchicine and with colchicine in combination with CCCP. n = 12 AMC; n = 21 colchicine; n = 10 colchicine+CCCP. p = 0.0005; Fisher exact test; multiple contingency.

(D) Membrane compliance of crests and grooves in AMC treated with colchicine and CCCP. n = 21 per group; **$p < 0.001$.

We wanted to test this on our model and disturbed microtubular polymerization with 10 μmol/l colchicine (Iribe et al., 2009; Figure 5). Colchicine didn't affect either the surface Z-groove architecture (Z-groove index; 0.61 ± 0.04 pre- versus 0.63 ± 0.05 post-treatment; p = ns; Figures S4A and S4B) or the T-tubular density (Figures S4C and S4D, left). However, both colchicine and nocodazole treatment significantly reduced T-tubule regularity (Figures S4C and S4D, right) and membrane compliance (Table S2). Having applied colchicine, we then investigated the frequency of propagated MiCa$_i$ with respect to location of the applied hydrojet pressure. Hydrojets applied to the crest of colchicine-treated cells initiated MiCa$_i$ in 69% of cases versus 12% in control AMC cells, i.e., without colchicine (Figure 5A). However, either combined colchicine plus CCCP (Figure 5C) or colchicine plus CsA (Figure S5A) treatments abolish this effect completely. Confocal and TEM microscopy show that colchicine displaces sub-sarcolemmal mitochondria in controls (Figure 5B, right panel) and the average mitochondrial area is increased, which makes them similar to the heart failure cells (Figures S6A, S6C, and S6D). Even in normal cells, colchicine stiffens the membrane (Figure 5D) and produces a similar membrane compliance to that seen in heart failure. This suggests that microtubular network dysregulation shifts the mitochondria, and that is the proposed critical mechanism underlying susceptibility to MiCa$_i$.

Previously, β-tubulin has been found to largely co-localize with cytoplasmic organelles, including mitochondria (Saetersdal et al., 1990). We therefore sought to investigate, in colchicine-treated cells, the relationship between the mitochondrial shift with its repositioning and the microtubular derangement. Immunocytochemical analysis demonstrated that colchicine significantly disrupted cardiomyocyte β-tubulin in both AMC cells (Figure 5B) and heart failure cells (Figure S6C). Supporting the notion that the tubulin network is distorted in heart failure cells, mRNA expression analysis by qPCR confirmed an overall increase in the expression of α1A-tubulin (*TUBA1A*), β2B-tubulin (*TUBB2B*), β3-tubulin (*TUBB3*), γ-1tubulin (*TUBG1*), and microtubule-associated proteins (*MAP4*; Figure S6D). These are known to be associated with altered microtubular dynamics (Roos et al., 2002). Interestingly, entirely disrupting the microtubular network in failing cardiomyocytes (colchicine administration) did not abolish MiCa$_i$ (Figure S7D) and significantly affected the membrane compliance (Figure S7C).

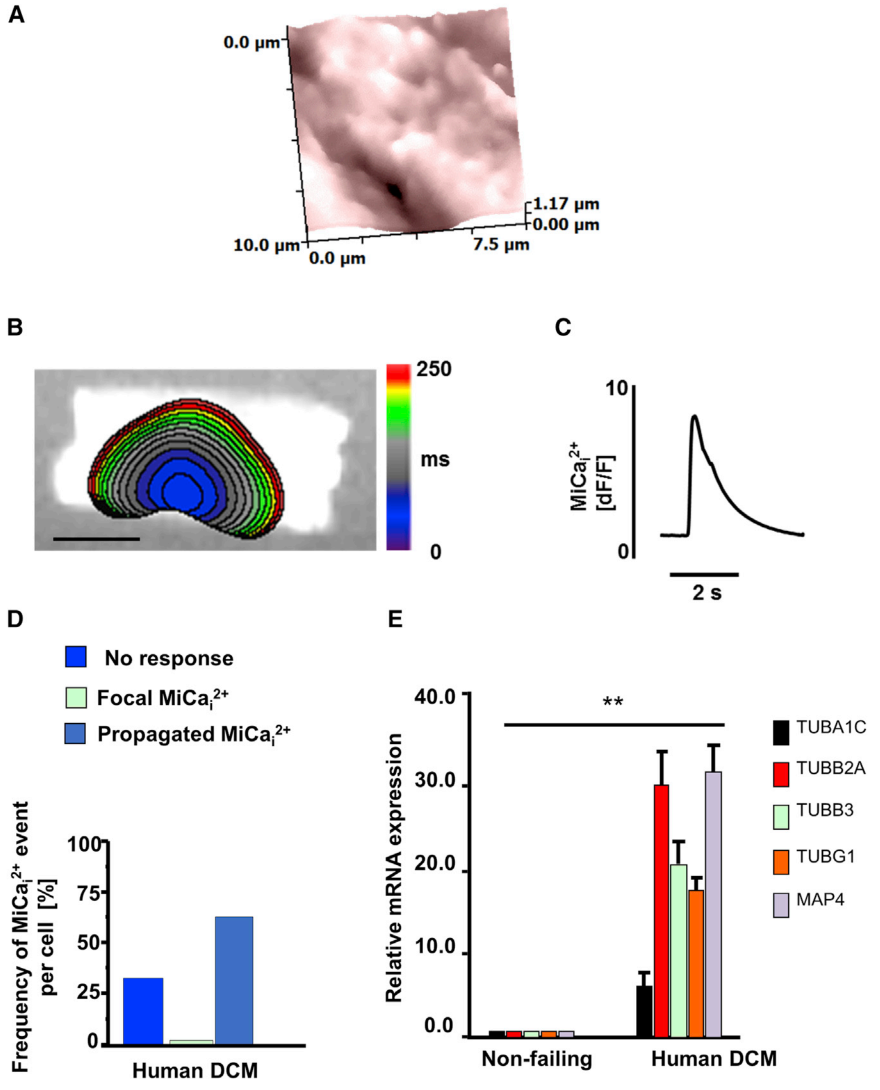

Figure 6. $MiCa_i$ Occurrence in Human DCM Cardiomyocytes

(A) Membrane topography of a human heart failure cardiomyocyte (10 × 10 µm).

(B) (Left-hand side) color-coded time-lapse map of $MiCa_i$ propagation. The scale bar represents 10 µm.

(C) Fluorescence trace of $MiCa_i$.

(D) Frequency of propagated $MiCa_i$ in human heart failure cells.

(E) Microtubule protein mRNA is upregulated in DCM cardiomyocytes as compared to non-failing human cardiomyocyte. Technical triplicate normalized to 18 s is shown. mRNA quantities are presented as mean ± SEM (**$p < 0.01$); n = 7.

$MiCa_i$ in Human Failing DCM Cells

We investigated the $MiCa_i$ incidence in human cardiomyocytes from dilated cardiomyopathy (DCM) patients. First, SICM imaging revealed a topographical heterogeneity (Figure 6A) similar to that previously seen in failing rat cardiomyocytes (Lyon et al., 2009). Propagating $MiCa_i$ (Figures 6B and 6C) occurred in 65% of all pressure applications, mainly when pressure was applied over non-striated, stiffer areas (Figure 6D), mimicking the rat heart failure model. Similarly to the rat failing cells, we found that, in human DCM cells, α1C-tubulin (*TUBA1C*), β2A-tubulin (*TUBB2A*), *TUBB3*, *TUBG1*, and *MAP4* were significantly upregulated as compared to non-failing cardiomyocytes, suggesting a primary role for microtubule disruption in cellular vulnerability to $MiCa_i$ generation (Figure 6E).

DISCUSSION

We report that a nanoscale perturbation of the surface membrane of control (AMC) and failing cardiomyocytes can elicit mitochondria-dependent Ca^{2+} release within milliseconds. This $MiCa_i$ is locally constrained and non-propagating. Sarcolemmal structural organization together with sub-sarcolemmal mitochondria regularity and high membrane compliance are prerequisite for impeding the $MiCa_i$ propagation (Figure 7; summarized in Table S2).

Mitochondria Implication in Mechanically Induced Calcium Initiation in Failing Cells

In healthy ventricular cardiomyocytes, sub-sarcolemmal mitochondria are arranged periodically under crests, separated by similarly periodic T-tubules. We suggest that this facilitates tight signal regulation with feedback loops close by, preventing calcium propagation along the sub-sarcolemma. The SICM distinguished crests and grooves on the surface of cardiomyocytes, and the same scanning nanopipette delivered hydrojets selectively to a nanoscaled area without damaging the membrane. This precise mechanical stimulation elicits a $MiCa_i$, which is normally constrained locally. In failing cardiomyocytes where the striations are progressively lost, particularly at 16 weeks post-MI, pressure application to a non-striated region triggered a propagated $MiCa_i$. Regular mitochondrial arrangement is lost in heart failure or experimentally induced microtubule derangement, implying that the microtubular network regulates the structural arrangement, with loss leading to altered restriction of membrane-perturbation-triggered $MiCa_i$, with a relatively large, propagating, expansive calcium response from mitochondria. Decreased membrane compliance is a prerequisite for eliciting $MiCa_i$. In heart failure, the membrane is stiffer, and this increases the likelihood of $MiCa_i$ (Borbély et al., 2005).

The mechanism implicates mitochondria, which are normally aligned under the crests and sense our applied force (in both non-failing and failing cells; Figure 7). Microtubules are pivotal

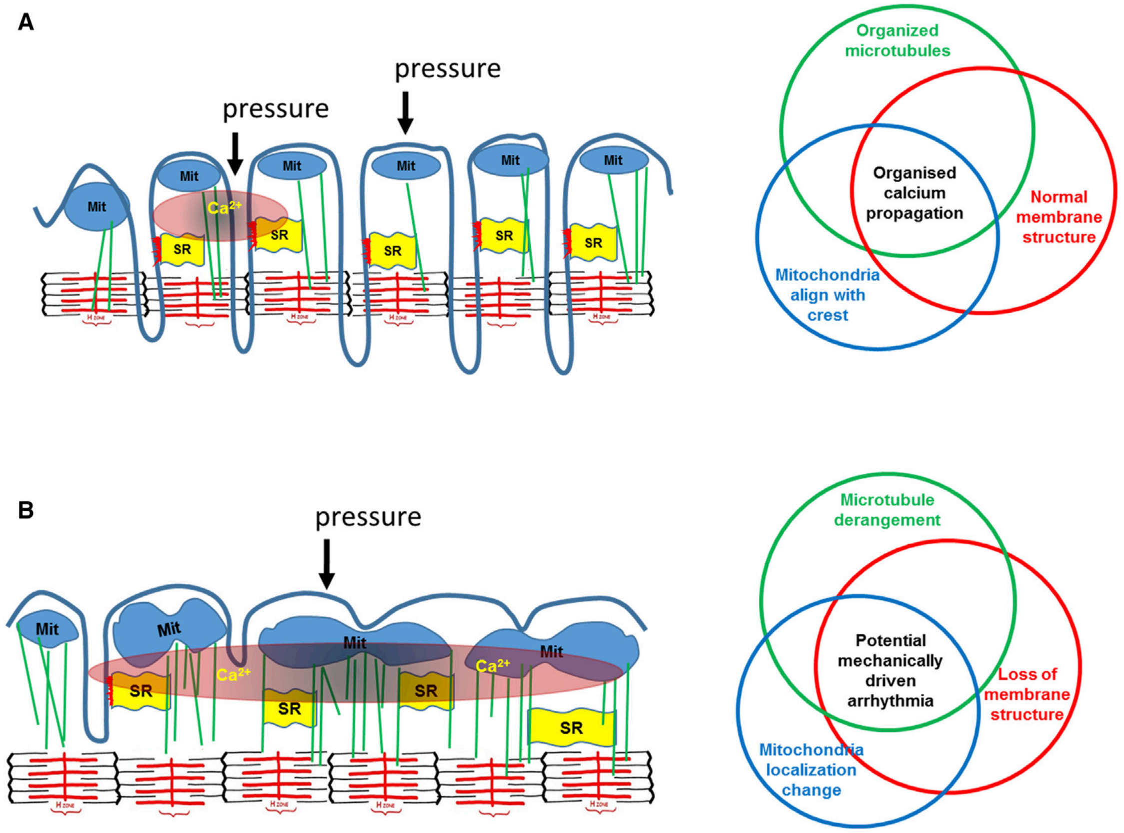

Figure 7. Schematic Representation of the Proposed Mechanisms of $MiCa_i$ Propagation

(A) Normal conditions. The interplay of an organized microtubular network, regular T-tubule membrane structure, and sub-sarcolemmal mitochondrial alignment protects against $MiCa_i$ propagation by providing tight control of calcium levels. MIT, mitochondria; SR, sarcoplasmic reticulum.

(B) Heart failure conditions. Overexpression and remodeling of microtubules together with mitochondrial delocalization and loss-of-membrane structural regularity enable $MiCa_i$ propagation due to loss of appropriate control.

in maintaining ordinary mitochondrial cytoarchitecture and in supporting physiological cellular membrane compliance.

Under Physiological Conditions, Higher Membrane Compliance Attenuates Forces Transmitted to the Mitochondria

We propose here that, in normal physiological conditions, higher membrane compliance (softer membrane) absorbs, attenuates, and buffers the forces transmitted to the mitochondria, whereas in heart failure cells, with microtubular derangement, lower membrane compliance (stiffer membrane) allows rapid force transmission to subcellular microdomains, which involve elongated and displaced mitochondria. The experimental data suggest that the remodeled mitochondria in failing cells are more sensitive to pressure changes within their microenvironment, producing an abnormal mitochondria Ca^{2+} release, which in turn triggers the calcium initiation and its propagation cascade. In line with this hypothesis, we show that pharmacological uncoupling of mitochondrial metabolism with CCCP abolishes $MiCa_i$ generation mainly because we introduce a break in the intracellular Ca^{2+}-driving source that can be mechanically activated via microtubular force transmission.

Mechanical Stimulation of Failing Cardiomyocytes Generates Multiple $MiCa_i$

In more-advanced stages of pathological remodeling 8–16 weeks following MI, we observed the generation of a second "ectopic" calcium wave that arises from remote regions of the cell. One plausible explanation is related to cross-bridge cycling. The local sarcomere contraction underneath the pressure site's $MiCa_i$ relaxes while more-distant sarcomeres contract, shortening against the lower compliance of the relaxing region. The shortening of these distant sarcomeres releases Ca^{2+} from its cycling troponin (Lab et al., 1984; ter Keurs et al., 2001) into the sarcoplasm. This could manifest as the remote peripheral Ca^{2+} signal. The other mechanism is that the stiffer sarcolemma transmits pressure to remote stretch-activated channels to admit calcium. Indeed, streptomycin or Gd^{3+} (selective blockers of stretch-activated channels) abolished this peripheral activation. Further investigations should provide further insight.

$MiCa_i$ propagation occurs in a single ripple initiation underneath the hydrojet pressure site. None of the following, stretch-activated channels inhibition by either streptomycin or Gd^{3+} or actin disruption with cytochalasin D, blocked both the initial

$MiCa_i$ signal and its propagation. In fact, 1 hr of cytochalasin D treatment in failing cells actually augments membrane stiffness and, by disrupting actin calcium-binding sites, abolishes contraction, despite leaving $MiCa_i$ initiation unaffected.

Microtubular Network Derangement Is a Prerequisite for $MiCa_i$ Propagation

Recent studies have implicated X-ROS signaling in inducing Ca^{2+} release (independently from mitochondria) by stretching the cell (Prosser et al., 2011). However, this activation required an intact microtubule network, which is supported by the observation that an increase in the microtubule network density (e.g., Duchenne muscular dystrophy; Prosser et al., 2013) decreases X-ROS signaling. We found that pharmacologically induced microtubular depolymerization in control cells produces a similar functional phenotype as that in heart failure cells, i.e., reduces membrane compliance and raises the likelihood of $MiCa_i$ propagation, which we see in 69% of cases (Figure 5A). Moreover, our intracellular findings agree with a large animal study, where colchicine exacerbated chest-impact-induced ventricular fibrillation (commotio cordis; Link et al., 1998). Upregulation of microtubular proteins encountered during heart failure (Roos et al., 2002) are implicated in destabilizing the microtubules network, by affecting T-tubule density and regularity. Disruption of the microtubule network allowed spreading of the normally constrained $MiCa_i$, mimicking the pattern of $MiCa_i$ propagation observed in failing cardiomyocytes. Mitochondria and microtubules are in intimate contact at sub-sarcolemmal levels because β-tubulin is confined to the perinuclear and inter-fibrillar spaces and is largely co-localized with the cytoplasmic organelles (Saetersdal et al., 1990). In cardiomyocytes, the distribution of β-tubulin-2 (Kuznetsov et al., 2013) is restricted to the outer mitochondrial-containing domain that binds to the outer mitochondrial membrane, and this probably also involves microtubular-based trans-locators and/or MAPs.

Sources of Calcium, Such as LTCCs and Sarcoplasmic Reticulum, Are Not Involved in $MiCa_i$ Generation

We did not find evidence that either extracellular calcium influx mediated by LTCCs or intracellular calcium from sarcoplasmic reticulum are important for mechanosensitive $MiCa_i$ generation and propagation. However, we cannot exclude that, after the initial calcium has been released from mitochondria, the additional calcium needed to produce a Ca^{2+} transient is released from the sarcoplasmic reticulum. This is because, in our experiments, we cannot fully deplete the sarcoplasmic reticulum of calcium, nor do we take into account the sarcoplasmic reticulum leak that loads the mitochondria with calcium (Santulli and Marks, 2015). However, mechanical stimulation may trigger a relatively small mitochondrial Ca^{2+} release to produce the sarcoplasmic reticulum Ca^{2+} release. This is because we still observe the $MiCa_i$ in low [Ca^{2+}] solution.

Microtubular Network Disorganization Provokes Mitochondria Displacement

Mitochondria re-locate from the crest during heart failure; the microtubule network disorganization modifies the mitochondria-crest interface and manifests as a reduction in the membrane compliance and decreased dyad regularity. Mitochondria relocation during heart failure, accompanied with increase in membrane stiffness, was recently described and investigated using atomic force microscopy (Dague et al., 2014). This study complements our data with our contact-free SICM and supports myocardial remodeling (with increasing average mitochondrial areas) with microtubular network derangement as crucial in the initiation of $MiCa_i$. Pressure-induced calcium release in a single cell is not a physiological issue as arrhythmia is a multicellular phenomenon; however, our proof of concept indicates microdomains disarray as an important mechanism. This, together with sarcomeric dyssynchrony in heart failure (Sachse et al., 2012), can lead to mechanically driven pathological consequences. Future challenges will be to investigate whether similar mechanisms are implicated in other cardiovascular pathologies such as hypertensive or ischemic heart disease.

Nonetheless, our observations have a potential translational element. Cellular heterogeneity flourishes in heart failure, and the mechanically induced calcium changes have electrophysiological consequences that are potentially arrhythmogenic. Approximately 15% of MI patients die from sustained ventricular tachycardia and fibrillation in the first 2 years after first hospitalization (Bloch Thomsen et al., 2010). The mechanisms we propose here can be included in the ''maladaptive electrical and mechanical remodeling,'' known to ultimately predispose the heart to arrhythmias by, for example., inducing calcium-overload-related triggered activity (Adamson et al., 2005; Wasson et al., 2004).

Conclusions

In summary, our combination of SICM and optical mapping of mechanically induced impulse propagation in a single cell is able to identify and localize functional mechanosensing with nanometer precision in live cells in general and, in particular, cardiomyocytes. We propose that, in heart-failure-derived cardiomyocytes, highly localized nanomechanical stress via their stiffer membranes and disrupted microtubule networks can trigger localized mitochondrial-dependent Ca^{2+} release, which initiates cell-wide Ca^{2+} wave propagation. As intracellular Ca^{2+} waves contribute to arrhythmogenesis on multicellular scales, the mechanisms we describe may represent an arrhythmogenic substrate for ectopic initiation and propagation. This not only provides mechanistic insights, it also provides new therapeutic targets. Moreover, changes in membrane compliance, which facilitate force transmission enabling calcium changes, could be a potential clinical marker.

EXPERIMENTAL PROCEDURES

Rat Cardiomyocytes Isolation

All animal surgical procedures and perioperative management conformed to the UK Animals (Scientific Procedures) Act 1986 of the Imperial College London Ethical Review Committee. The project license authorized these studies in accordance with the United Kingdom Home Office Animals (Scientific Procedures) Act 1986. Adult male Sprague-Dawley rats (250–300 g) underwent proximal left anterior descending coronary ligation to induce chronic MI as described previously (Lyon et al., 2009; see Supplemental Information for

details). Only cardiomyocytes from ventricular zones remote from the scar were utilized for the experiments.

Human Cardiomyocytes Isolation

Human myocardium was obtained from explanted hearts of patients with DCM undergoing cardiac transplantation with the approval from Bromton Harefield and NHLI Research Ethic Committee (ref 01-194). Human cardiomyocytes were isolated, macerating tissue and incubating sequentially in low-calcium- and collagenase/protease-containing solutions as described previously (del Monte et al., 1999).

In Vivo Cardiac Function

Cardiac function was assessed via biometrics and echocardiography. Heart weight corrected to tibia length provided a measure of hypertrophy. Echocardiography was performed under general anesthesia (2% isoflurane) immediately prior to dispatch to give a measure of in vivo cardiac function. The imaging was performed in M-mode in the parasternal long axis view (Table S1; Vevo 770 system). After 4, 8, or 16 weeks following coronary ligation, rats were dispatched by cervical dislocation after brief exposure to 5% isoflurane until the righting reflex was lost. We perfused the left ventricle via the Langendorff perfusion apparatus (Sato et al., 2005). Cardiomyocytes were enzymatically isolated from the left ventricle.

Customization of Hopping Probe SICM and Optical Mapping of Impulse Propagation

The SICM setup has been previously described (Miragoli et al., 2011), as has its application in the hopping mode (Novak et al., 2009). We combined this system with a fast, high-resolution optical camera (Ultima-Scimedia) mounted on an inverted microscope. This was focused upon the membrane region subtending the scanning pipette of the SICM (Figure 1). Briefly, a piezo-controller (ICnano Scanner Controller; Ionscope) controlled the xyz piezo three-axis translation stage Triton-100 (Piezosystem) with 80-μm closed-loop travel range in x, y, and z directions. The piezo stage was driven by high-voltage amplifier System ENV 150 (Piezosystem) connected to ICnano scanner controller. The pipette electrode head stage was connected to Multiclamp 700B (Molecular Devices). The scan head was placed on the platform of Nikon TE-i inverted microscope (Nikon Corporation). Nanopipettes (~25–100 MΩ tip resistance) were pulled from borosilicate glass (O.D. 1.0 mm; I.D. 0.58 mm; Intracell) using a laser puller (P-2000; Sutter) The pipettes were filled with Hanks' balanced salt solution (HBSS) and utilized to acquire SICM images of membrane topography. These were utilized for all the hydrojet pressure-application experiments (see Supplemental Information for details).

Hydrojet Pressure Application

A square pulse or ramp (depending on the experiment) of air pressure delivered by displacing air connected to the auxiliary inlet in the pipette holder generated a hydrojet of the intracellular pipette solution (HBSS); the delivery was controlled by an electric valve via Digidata 1440A (Molecular Devices). We choose 20 kPa for a square pulse pressure, resulting in a perturbation of ~0.125 μm^2 area within the sarcomere. Membrane compliance was derived from the nanopipette's vertical displacement (Z-direction), acquired using pClamp 10.0 (Molecular Devices). The volumetric flow rate and the hydrojet velocity were calculated as previously described (Sánchez et al., 2008).

Surface Confocal SICM Study of Mitochondria

We imaged mitochondria in control cells and those 16 weeks post-MI. Cells were stained with the fluorescent tetramethylrhodamine methyl ester (TMRM) dye (mitochondrial membrane potential dye) for 10 min at 37°C (100 nmol/l). TMRM was excited at 532 nm with a MLL532 20-mV laser (Changchun New Industries Optoelectronics Tech), and confocal images were taken at 100× magnification using a Photomultiplier Detection System (PTI). We aligned the laser beam with the tip of the pipette, then we scanned the cell surface with the SICM to obtain a topographical image, and finally we re-scanned the same area with the confocal laser beam to visualize the mitochondria. The two resulting images were overlaid to co-localize the mitochondria with cellular topography.

Optical Mapping of Intracellular Impulse Propagation

After isolation, cardiomyocytes were seeded onto 22-mm coverslips, loaded with 5 μmol/l Fluo-4 AM (Invitrogen) as an intracellular $[Ca^{2+}]_i$ transient indicator and incubated for 20 min at 35°C, 5% CO_2 before mounting on a customized perfusion chamber. $[Ca^{2+}]_i$ transients were acquired at 1–10 kHz sample resolution at 36°C, using a 40× objective with a fast resolution CMOS camera (Ultima; Scimedia) and dedicated acquisition software (MiCam Brainvision). A dedicated piece of software (Brainvision Ana v. 1208) was utilized to determine intracellular calcium propagation.

Synchronization of the Acquisition

A protocol written in pClamp 10.0 was used to synchronize the pressure application and the optical calcium recording via Digidata 1440A (Molecular Devices). It synchronized triggering the open/closed states of the electric valve (2 s; square pulse) and the light shutter (Uniblitz; Vincent Associated). Each recording lasted 8 s.

Drug Dilutions

Caffeine (10 mmol/l), colchicine (10 μmol/l), nocodazole (4 μg/ml), streptomycin (100 μmol/l), nifedipine (2 μmol/l), carbonyl cyanide *m*-chlorophenyl hydrazine (CCCP) (0.1 μmol/l), and CsA (0.5 μmol/l) were dissolved in HBSS containing (in mmol/l) NaCl (144), HEPES (10), $MgCl_2$ (1), and KCl (5; 0.1 mmol/l $CaCl_2$ for CCCP and colchicine experiments). Cytochalasin D was dissolved in DMSO and diluted in HBSS. Gadolinium (Gd^{3+}) was dissolved in miliQ water and diluted to 30 μM in HBSS immediately before use (Yeung et al., 2003).

T-Tubule Density and Regularity Measurements

Left ventricle cardiomyocytes were derived from control animals and those 16 weeks post-MI. Cells were stained with the fluorescent dye Di-8-ANEPPS (10 μmol/l) for 1 min. Di-8-ANEPPS was excited at a wavelength of 488 nm, and confocal z-stacked images were taken at 63× magnification using a LSM-780 inverted confocal microscope (Zeiss). The resulting images of the T-tubule network were analyzed using the freeware ImageJ (http://rsbweb.nih.gov/ij/) by randomly choosing two separate areas of 40 × 5 microns. The chosen areas were converted into binarized black and white images and then plotted into waveforms. The binarized versions of the confocal images were used to define the T-tubule density by calculating the ratio of black to white pixels in each chosen area whereas the waveforms were transformed into power-frequency peaks through a 1D Fourier transformation using a custom-written macro for the software Matlab (The MathWorks). The amplitudes of the calculated peaks were plotted as T-tubule regularity and were interpreted as an indicator of how regular T-tubules appear.

Confocal Microscopy Images of Mitochondria and T-Tubules

Control cells (AMC), MI-16 weeks, and AMC cells treated with colchicine were stained as previously described with TMRM and DI-8-ANEPPS. Confocal z-stacked images were taken at 63× magnification using a Zeiss LSM-780 inverted confocal microscope. The AMC cells were treated with colchicine (10 μmol/l) for 3 hr as known to be adequate for the selective disruption of microtubules (White, 2011). The resultant images of the T-tubule network and mitochondria were overlaid using the freeware ImageJ software.

Analysis of Confocal Microscopy Images of Mitochondria

The resulting images of the mitochondria were analyzed using the freeware program ImageJ (http://rsbweb.nih.gov/ij/) with a mitochondrial morphology plugin (Dagda et al., 2009). Three slices from the z stack were analyzed for each cell to obtain an average area of the mitochondria elements per cell. In brief, after selecting the area of the cell to be analyzed, avoiding the edge of the cells, the image is binarized, and the plugin measures the area for each single element (single mitochondria and cluster of mitochondria).

Data Statistics

All data are described as mean ± SEM for the given number of experiments. Significance was calculated using Student's t test and Fisher exact tests and is indicated in the figure legends.

OPEN
ACCESS
CellPress

SUPPLEMENTAL INFORMATION

Supplemental Information includes Supplemental Experimental Procedures, seven figures, two tables, and three movies and can be found with this article online at http://dx.doi.org/10.1016/j.celrep.2015.12.014.

AUTHOR CONTRIBUTIONS

M.M., M.J.L., and J.G. initiated the study. M.M. performed all the pressure application, optical mapping, and membrane compliance experiments and analyzed the data associated with it. J.L.S.-A. conducted the SICM surface confocal and the TMRM confocal images and analyzed the data associated with it. I.D. performed the TEM and the tubulin staining and analyzed the data associated with it. A.B. conducted the SICM experiments of cardiomyocytes and z-groove calculations. P.T.W. isolated the human cardiomyocytes. M.S. and A.R.L. generated the heart failure model. S.S. performed the TT density and regularity measurements. P.N. helped with SICM adaptation for membrane compliance. A.C. and P.C. performed the mRNA studies. M.M., J.G., and M.J.L. conceived and designed research, performed data analysis, and wrote the manuscript. All authors discussed and contributed to the manuscript.

ACKNOWLEDGMENTS

We are grateful to Prof. Cristobal dos Remedios, University Sydney, and Prof. Steve Marston for human heart muscle samples as well as Mr. Peter O'Gara for cell isolation. We thank Andrew Rogers from the Royal Brompton and Harefield NHS Foundation Trust Lung Pathology Unit, Royal Brompton Hospital, for help with transmission electron microscopy. This study was supported by Wellcome Trust (WT090594 to J.G. and WT092852 to M.S.), British Heart Foundation (12/18/30088 to J.G.), Italian Ministry of Health (GR-2009-1530528 to M.M.), BHF Intermediate Research Fellowship (FS/11/67/28954 to A.R.L.), and Graham Dixon Charitable Trust (registered charity number: 277057).

Received: April 21, 2015
Revised: September 7, 2015
Accepted: November 23, 2015
Published: December 24, 2015

REFERENCES

Adamson, P.B., Barr, R.C., Callans, D.J., Chen, P.-S., Lathrop, D.A., Makielski, J.C., Nerbonne, J.M., Nuss, H.B., Olgin, J.E., Przywara, D.A., et al. (2005). The perplexing complexity of cardiac arrhythmias: beyond electrical remodeling. Heart Rhythm *2*, 650–659.

Belmonte, S., and Morad, M. (2008a). 'Pressure-flow'-triggered intracellular Ca2+ transients in rat cardiac myocytes: possible mechanisms and role of mitochondria. J. Physiol. *586*, 1379–1397.

Belmonte, S., and Morad, M. (2008b). Shear fluid-induced Ca2+ release and the role of mitochondria in rat cardiac myocytes. Ann. N Y Acad. Sci. *1123*, 58–63.

Bloch Thomsen, P.E., Jons, C., Raatikainen, M.J., Moerch Joergensen, R., Hartikainen, J., Virtanen, V., Boland, J., Anttonen, O., Gang, U.J., Hoest, N., et al.; Cardiac Arrhythmias and Risk Stratification After Acute Myocardial Infarction (CARISMA) Study Group (2010). Long-term recording of cardiac arrhythmias with an implantable cardiac monitor in patients with reduced ejection fraction after acute myocardial infarction: the Cardiac Arrhythmias and Risk Stratification After Acute Myocardial Infarction (CARISMA) study. Circulation *122*, 1258–1264.

Borbély, A., van der Velden, J., Papp, Z., Bronzwaer, J.G., Edes, I., Stienen, G.J., and Paulus, W.J. (2005). Cardiomyocyte stiffness in diastolic heart failure. Circulation *111*, 774–781.

Borg, T.K., Goldsmith, E.C., Price, R., Carver, W., Terracio, L., and Samarel, A.M. (2000). Specialization at the Z line of cardiac myocytes. Cardiovasc. Res. *46*, 277–285.

Chen, Y., Csordás, G., Jowdy, C., Schneider, T.G., Csordás, N., Wang, W., Liu, Y., Kohlhaas, M., Meiser, M., Bergem, S., et al. (2012). Mitofusin 2-containing mitochondrial-reticular microdomains direct rapid cardiomyocyte bioenergetic responses via interorganelle Ca(2+) crosstalk. Circ. Res. *111*, 863–875.

Dagda, R.K., Cherra, S.J., 3rd, Kulich, S.M., Tandon, A., Park, D., and Chu, C.T. (2009). Loss of PINK1 function promotes mitophagy through effects on oxidative stress and mitochondrial fission. J. Biol. Chem. *284*, 13843–13855.

Dague, E., Genet, G., Lachaize, V., Guilbeau-Frugier, C., Fauconnier, J., Mias, C., Payré, B., Chopinet, L., Alsteens, D., Kasas, S., et al. (2014). Atomic force and electron microscopic-based study of sarcolemmal surface of living cardiomyocytes unveils unexpected mitochondrial shift in heart failure. J. Mol. Cell. Cardiol. *74*, 162–172.

del Monte, F., Harding, S.E., Schmidt, U., Matsui, T., Kang, Z.B., Dec, G.W., Gwathmey, J.K., Rosenzweig, A., Hajjar, R.J., and Hajjar, R.J. (1999). Restoration of contractile function in isolated cardiomyocytes from failing human hearts by gene transfer of SERCA2a. Circulation *100*, 2308–2311.

Dobrev, D., and Wehrens, X.H. (2014). Role of RyR2 phosphorylation in heart failure and arrhythmias: Controversies around ryanodine receptor phosphorylation in cardiac disease. Circ. Res. *114*, 1311–1319, discussion 1319.

Ermakov, Y.A., Kamaraju, K., Sengupta, K., and Sukharev, S. (2010). Gadolinium ions block mechanosensitive channels by altering the packing and lateral pressure of anionic lipids. Biophys. J. *98*, 1018–1027.

Ferreira-Cornwell, M.C., Luo, Y., Narula, N., Lenox, J.M., Lieberman, M., and Radice, G.L. (2002). Remodeling the intercalated disc leads to cardiomyopathy in mice misexpressing cadherins in the heart. J. Cell Sci. *115*, 1623–1634.

Ingber, D.E. (1997). Tensegrity: the architectural basis of cellular mechanotransduction. Annu. Rev. Physiol. *59*, 575–599.

Iribe, G., Ward, C.W., Camelliti, P., Bollensdorff, C., Mason, F., Burton, R.A., Garny, A., Morphew, M.K., Hoenger, A., Lederer, W.J., and Kohl, P. (2009). Axial stretch of rat single ventricular cardiomyocytes causes an acute and transient increase in Ca2+ spark rate. Circ. Res. *104*, 787–795.

Janmey, P.A., and Miller, R.T. (2011). Mechanisms of mechanical signaling in development and disease. J. Cell Sci. *124*, 9–18.

Kim, S.J., Iizuka, K., Kelly, R.A., Geng, Y.J., Bishop, S.P., Yang, G., Kudej, A., McConnell, B.K., Seidman, C.E., Seidman, J.G., and Vatner, S.F. (1999). An alpha-cardiac myosin heavy chain gene mutation impairs contraction and relaxation function of cardiac myocytes. Am. J. Physiol. *276*, H1780–H1787.

Kiseleva, I., Kamkin, A., Wagner, K.D., Theres, H., Ladhoff, A., Scholz, H., Günther, J., and Lab, M.J. (2000). Mechanoelectric feedback after left ventricular infarction in rats. Cardiovasc. Res. *45*, 370–378.

Knöll, R., Hoshijima, M., Hoffman, H.M., Person, V., Lorenzen-Schmidt, I., Bang, M.L., Hayashi, T., Shiga, N., Yasukawa, H., Schaper, W., et al. (2002). The cardiac mechanical stretch sensor machinery involves a Z disc complex that is defective in a subset of human dilated cardiomyopathy. Cell *111*, 943–955.

Kohlhaas, M., and Maack, C. (2013). Calcium release microdomains and mitochondria. Cardiovasc. Res. *98*, 259–268.

Kuznetsov, A.V., Javadov, S., Guzun, R., Grimm, M., and Saks, V. (2013). Cytoskeleton and regulation of mitochondrial function: the role of beta-tubulin II. Front. Physiol. *4*, 82.

Lab, M.J., Allen, D.G., and Orchard, C.H. (1984). The effects of shortening on myoplasmic calcium concentration and on the action potential in mammalian ventricular muscle. Circ. Res. *55*, 825–829.

Lammerding, J., Kamm, R.D., and Lee, R.T. (2004). Mechanotransduction in cardiac myocytes. Ann. N Y Acad. Sci. *1015*, 53–70.

Link, M.S., Wang, P.J., Pandian, N.G., Bharati, S., Udelson, J.E., Lee, M.Y., Vecchiotti, M.A., VanderBrink, B.A., Mirra, G., Maron, B.J., and Estes, N.A., 3rd. (1998). An experimental model of sudden death due to low-energy chest-wall impact (commotio cordis). N. Engl. J. Med. *338*, 1805–1811.

Lu, X., Ginsburg, K.S., Kettlewell, S., Bossuyt, J., Smith, G.L., and Bers, D.M. (2013). Measuring local gradients of intramitochondrial [Ca(2+)] in cardiac myocytes during sarcoplasmic reticulum Ca(2+) release. Circ. Res. *112*, 424–431.

Lyon, A.R., MacLeod, K.T., Zhang, Y., Garcia, E., Kanda, G.K., Lab, M.J., Korchev, Y.E., Harding, S.E., and Gorelik, J. (2009). Loss of T-tubules and other changes to surface topography in ventricular myocytes from failing human and rat heart. Proc. Natl. Acad. Sci. USA *106*, 6854–6859.

Lyon, A.R., Nikolaev, V.O., Miragoli, M., Sikkel, M.B., Paur, H., Benard, L., Hulot, J.S., Kohlbrenner, E., Hajjar, R.J., Peters, N.S., et al. (2012). Plasticity of surface structures and β(2)-adrenergic receptor localization in failing ventricular cardiomyocytes during recovery from heart failure. Circ Heart Fail *5*, 357–365.

Miragoli, M., Moshkov, A., Novak, P., Shevchuk, A., Nikolaev, V.O., El-Hamamsy, I., Potter, C.M., Wright, P., Kadir, S.H., Lyon, A.R., et al. (2011). Scanning ion conductance microscopy: a convergent high-resolution technology for multi-parametric analysis of living cardiovascular cells. J. R. Soc. Interface *8*, 913–925.

Novak, P., Li, C., Shevchuk, A.I., Stepanyan, R., Caldwell, M., Hughes, S., Smart, T.G., Gorelik, J., Ostanin, V.P., Lab, M.J., et al. (2009). Nanoscale live-cell imaging using hopping probe ion conductance microscopy. Nat. Methods *6*, 279–281.

Piquereau, J., Caffin, F., Novotova, M., Lemaire, C., Veksler, V., Garnier, A., Ventura-Clapier, R., and Joubert, F. (2013). Mitochondrial dynamics in the adult cardiomyocytes: which roles for a highly specialized cell? Front. Physiol. *4*, 102.

Prosser, B.L., Ward, C.W., and Lederer, W.J. (2011). X-ROS signaling: rapid mechano-chemo transduction in heart. Science *333*, 1440–1445.

Prosser, B.L., Khairallah, R.J., Ziman, A.P., Ward, C.W., and Lederer, W.J. (2013). X-ROS signaling in the heart and skeletal muscle: stretch-dependent local ROS regulates $[Ca^{2+}]$i. J. Mol. Cell. Cardiol. *58*, 172–181.

Roos, K.P., Palmer, R.E., and Miller, T.W. (2002). The role of microtubules in structural remodeling and the progression to heart failure. J. Card. Fail. *8* (*6, Suppl*), S300–S310.

Rosca, M.G., Tandler, B., and Hoppel, C.L. (2013). Mitochondria in cardiac hypertrophy and heart failure. J. Mol. Cell. Cardiol. *55*, 31–41.

Sachse, F.B., Torres, N.S., Savio-Galimberti, E., Aiba, T., Kass, D.A., Tomaselli, G.F., and Bridge, J.H. (2012). Subcellular structures and function of myocytes impaired during heart failure are restored by cardiac resynchronization therapy. Circ. Res. *110*, 588–597.

Saetersdal, T., Greve, G., and Dalen, H. (1990). Associations between beta-tubulin and mitochondria in adult isolated heart myocytes as shown by immunofluorescence and immunoelectron microscopy. Histochemistry *95*, 1–10.

Sánchez, D., Johnson, N., Li, C., Novak, P., Rheinlaender, J., Zhang, Y., Anand, U., Anand, P., Gorelik, J., Frolenkov, G.I., et al. (2008). Noncontact measurement of the local mechanical properties of living cells using pressure applied via a pipette. Biophys. J. *95*, 3017–3027.

Santulli, G., and Marks, A.R. (2015). Essential roles of intracellular calcium release channels in muscle, brain, metabolism, and aging. Curr. Mol. Pharmacol. *8*, 206–222.

Sato, M., O'Gara, P., Harding, S.E., and Fuller, S.J. (2005). Enhancement of adenoviral gene transfer to adult rat cardiomyocytes in vivo by immobilization and ultrasound treatment of the heart. Gene Ther. *12*, 936–941.

ter Keurs, H.E., Zhang, Y.M., Davidoff, A.W., Boyden, P.A., Wakayama, Y., and Miura, M. (2001). Damage induced arrhythmias: mechanisms and implications. Can. J. Physiol. Pharmacol. *79*, 73–81.

Undrovinas, A.I., and Maltsev, V.A. (1998). Cytochalasin D alters kinetics of Ca2+ transient in rat ventricular cardiomyocytes: an effect of altered actin cytoskeleton? J. Mol. Cell. Cardiol. *30*, 1665–1670.

Wasson, S., Reddy, H.K., and Dohrmann, M.L. (2004). Current perspectives of electrical remodeling and its therapeutic implications. J. Cardiovasc. Pharmacol. Ther. *9*, 129–144.

White, E. (2006). Mechanosensitive channels: therapeutic targets in the myocardium? Curr. Pharm. Des. *12*, 3645–3663.

White, E. (2011). Mechanical modulation of cardiac microtubules. Pflugers Arch. *462*, 177–184.

Yeung, E.W., Head, S.I., and Allen, D.G. (2003). Gadolinium reduces short-term stretch-induced muscle damage in isolated mdx mouse muscle fibres. J. Physiol. *552*, 449–458.

Cell Reports
Supplemental Information

Microtubule-Dependent Mitochondria Alignment Regulates Calcium Release in Response to Nanomechanical Stimulus in Heart Myocytes

Michele Miragoli, Jose L. Sanchez-Alonso, Anamika Bhargava, Peter T. Wright, Markus Sikkel, Sophie Schobesberger, Ivan Diakonov, Pavel Novak, Alessandra Castaldi, Paola Cattaneo, Alexander R. Lyon, Max J. Lab, and Julia Gorelik

Supplemental Information

Supplemental figures.

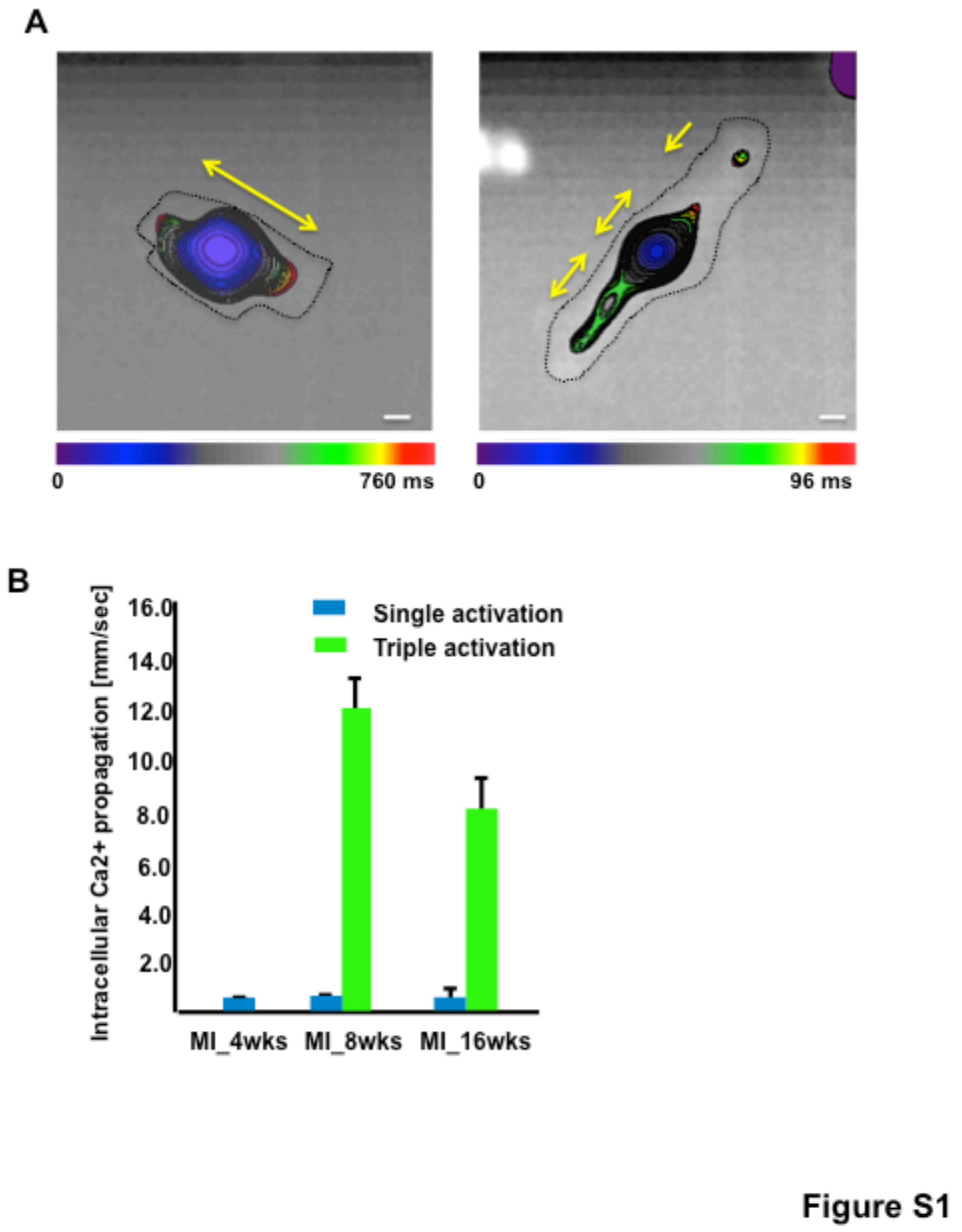

Figure S1, related to Figure 3

Propagation of MiCa$_i$ often evokes initiation of secondary calcium waves at the cell edges. A. color-coded time-lapse map of MiCa$_i$ with either single (left) or triple (right) initiation. Bar= 10 μ **B**. Intracellular calcium propagation velocity at different times following MI.

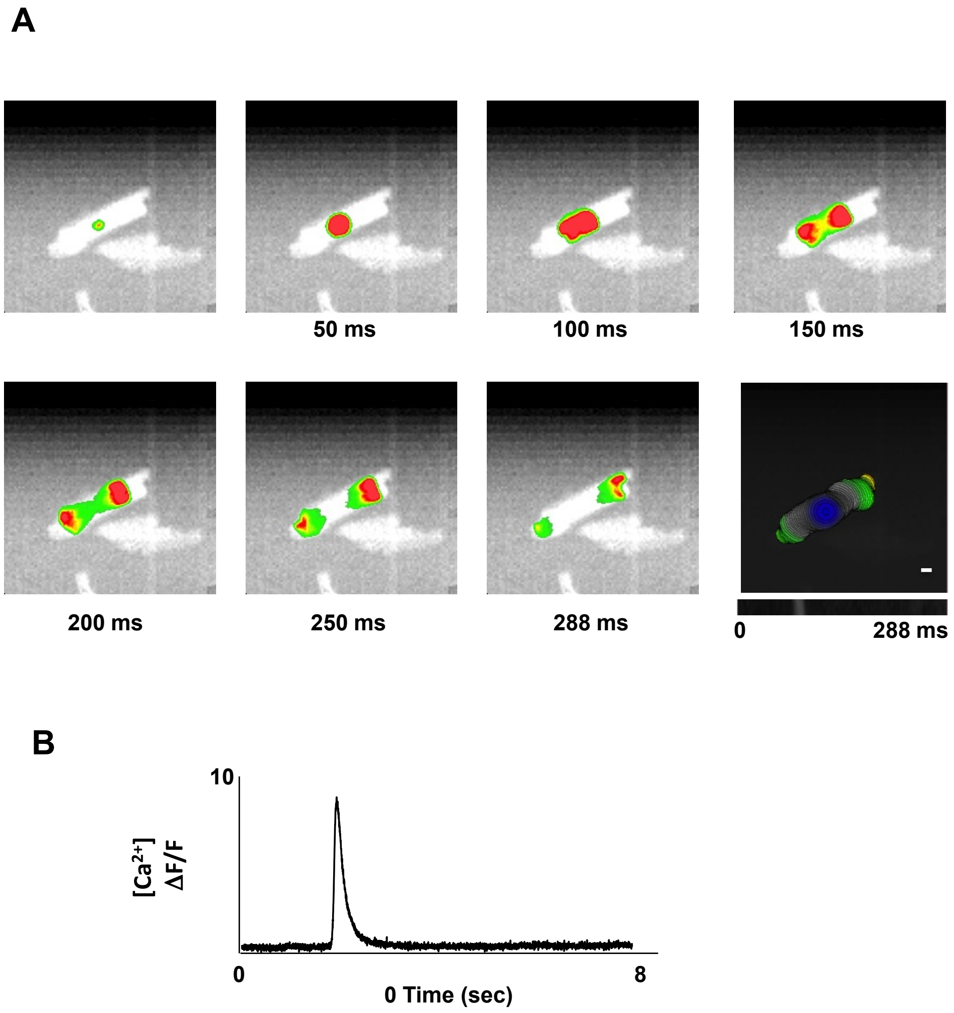

Figure S2

Figure S2, related to Figure 3

Inhibition of mechanosensitive ion channels does not stop MiCa$_i$ initiation. The cardiomyocytes were exposed to 30 μmol/L gadolinium. **A**. Representative frames (time interval 50 ms) of MiCa$_i$ initiated at the pressure site (upper left) and propagated as ripple effect toward the cell edges; bottom right: isochronic color-coded map). **B**. MiCa$_i$ trace for the cardiomyocyte exposed to gadolinium shown in A. Scale bar 10 μm, n=6.

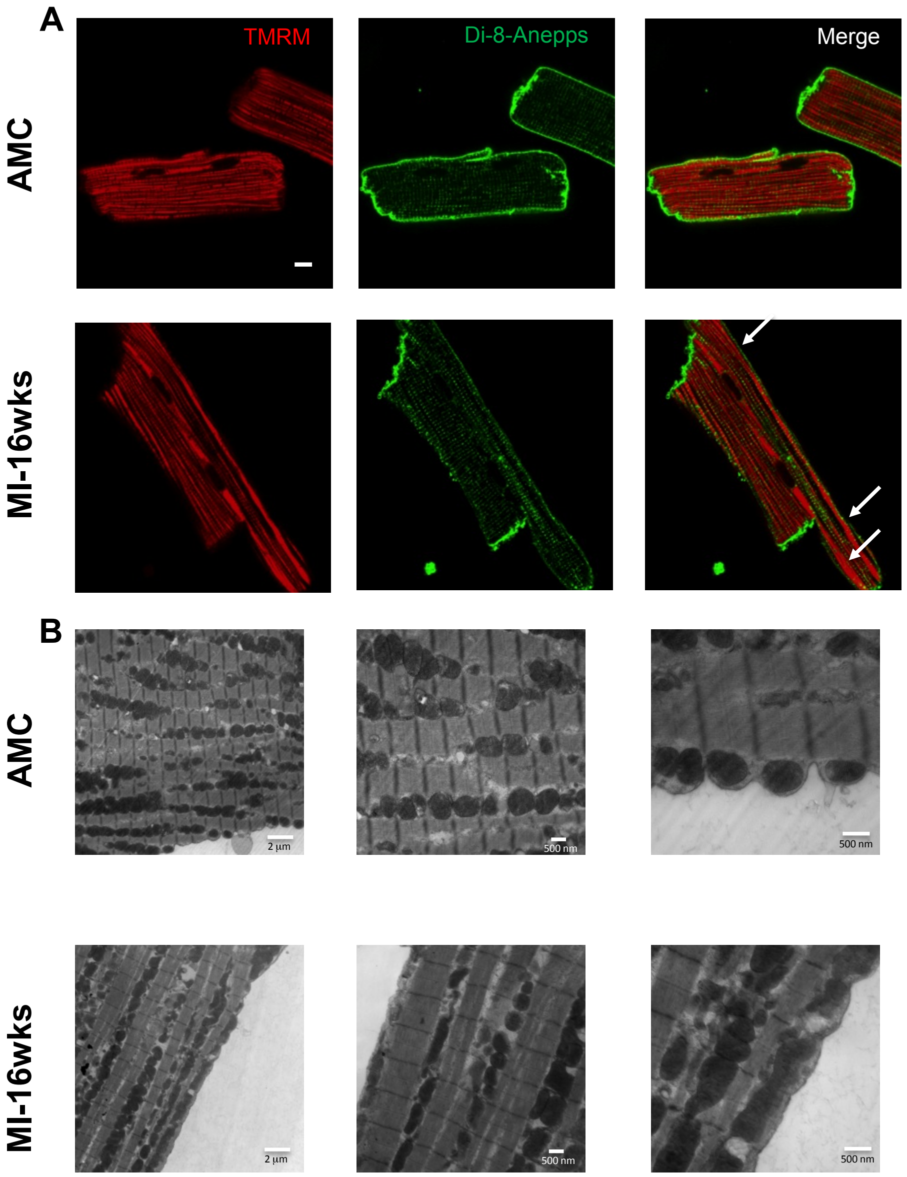

Figure S3

Figure S3, related to Figure 4

Remodelling of mitochondria and T-tubule network following MI. A. Mitochondria are stained with TMRM; T-tubules are stained with Di-8-ANEPPS in AMC myocytes (top row) and failing cells (bottom row). Bar= 10 µm. Arrows indicate mitochondria enlargement and relocalization. **B**. Mitochondria organization displayed in TEM images for control caridoymocytes (AMC, top) and failing cardiomyocytes (HF, bottom) showing mitochondrial enlargement at different magnification (from left to right 5000X, 10000X, 20000X).

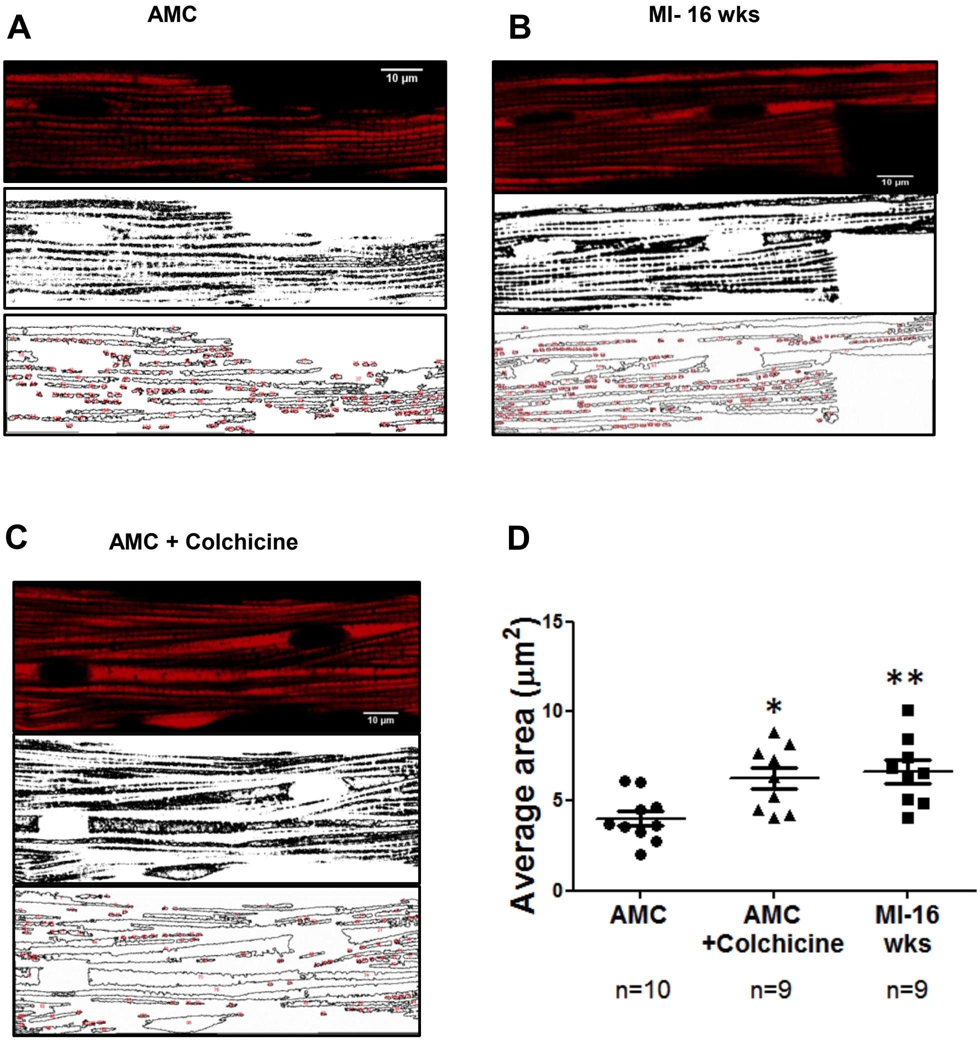

Figure S4

Figure S4, related to Figure 4

Estimation of mitochondrial elongation. A. Top: confocal image of a normal cardiomyocyte with mitochondria labeled (TMRM). Middle: the same image binarized. Bottom: regions of interest automatically selected for area calculations by a plug-in in ImageJ software. **B**. Same as A for a control cardiomyocyte exposed to colchicine. **C**. Same as A for a heart failure cardiomyocyte. **D**. Analysis of mitochondrial area in the three conditions: control, control+colchicine and heart failure. n>9. P<0.05.

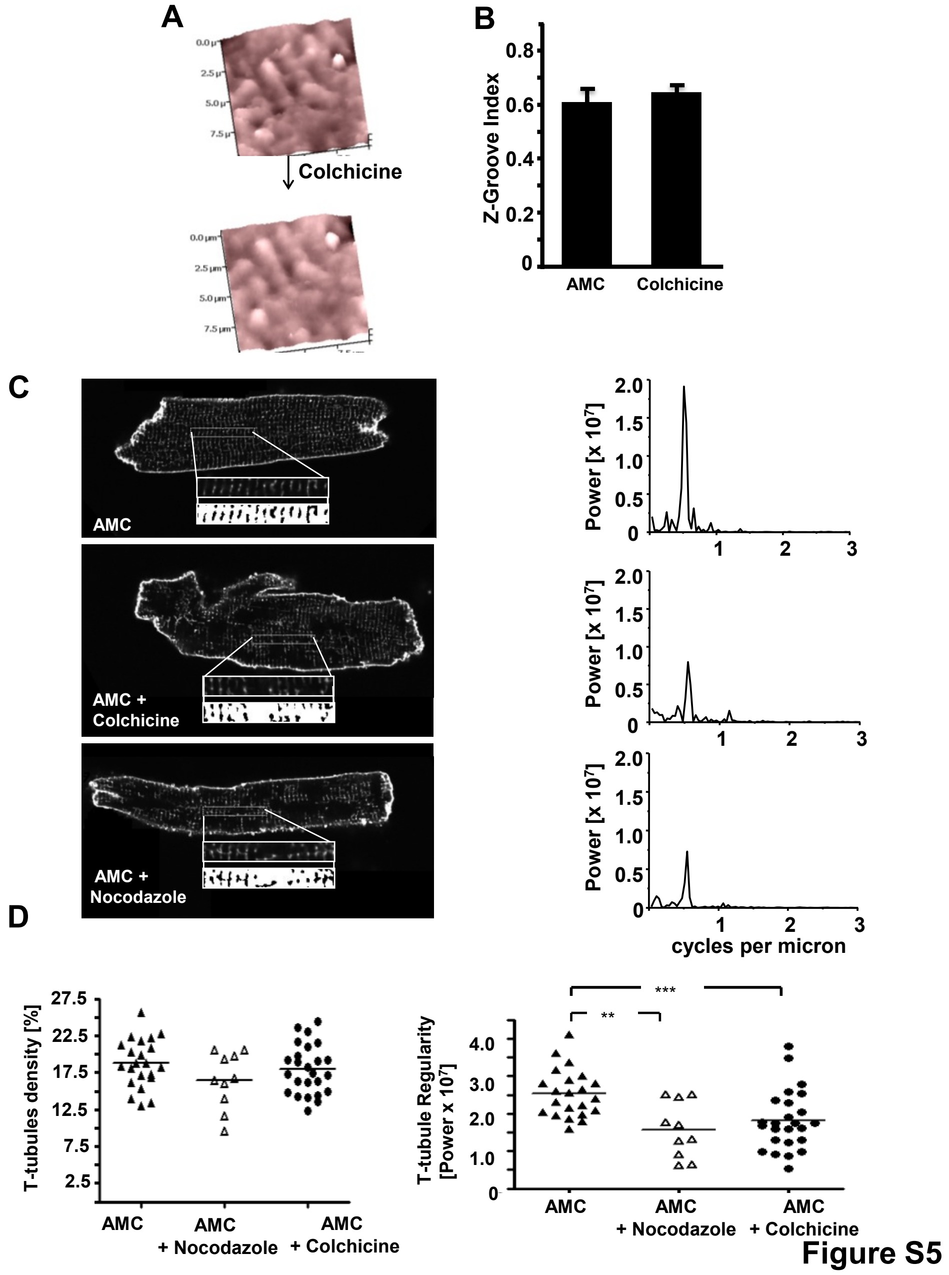

Figure S5, related to Figure 5

Effect of Colchicine on T-tubules and Z-grooves A. Topography of an AMC cardiomyocyte after treatment with colchicine (10µmol/l). **B.** Z-groove index after colchicine treatment does not change; **C.** Left: T-tubule staining with Di-8-ANEPPS after colchicine or nocodazole treatment. Insets: binarised images of selected areas on which regularity calculations have been made; Right: Power of regularity of intensity peaks corresponding to the images on the left (fast Fourier Transform analysis); **D.** T-tubule density (left panel) and regularity (right panel) after colchicine or nocodazole treatment.

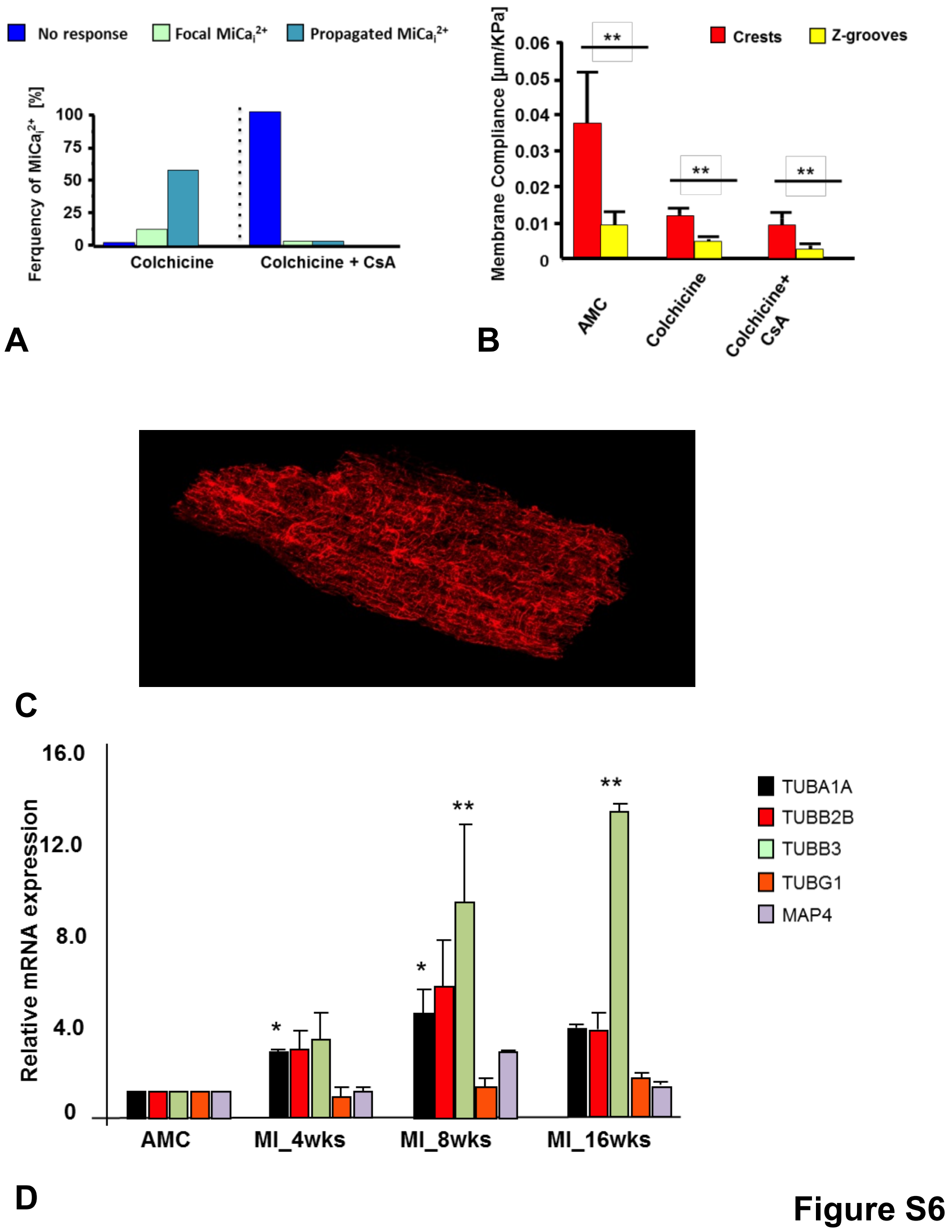

Figure S6

Figure S6, related to Figure 3 and 5

Cyclosporine A abolishes MiCa$_i$ after colchicine treatment but does not affect membrane compliance. A. Occurrence of MiCa$_i$ events in control cells subjected to colchicine and colchicine plus CsA. **B**. Membrane compliance of Z-grooves and crests in control cells, control cells subjected to colchicine and to colchicine plus CsA.

Changes in tubulin expression in HF. C. Representative image of a cardiomyocyte at 16weeks post-MI stained for β-tubulin. Scale bar is 10 µm. **D**. Relative mRNA expression for various microtubule proteins in: age matched control AMC, and different times following MI. (n=3 x technical triplicate normalized against Cyclophillin).

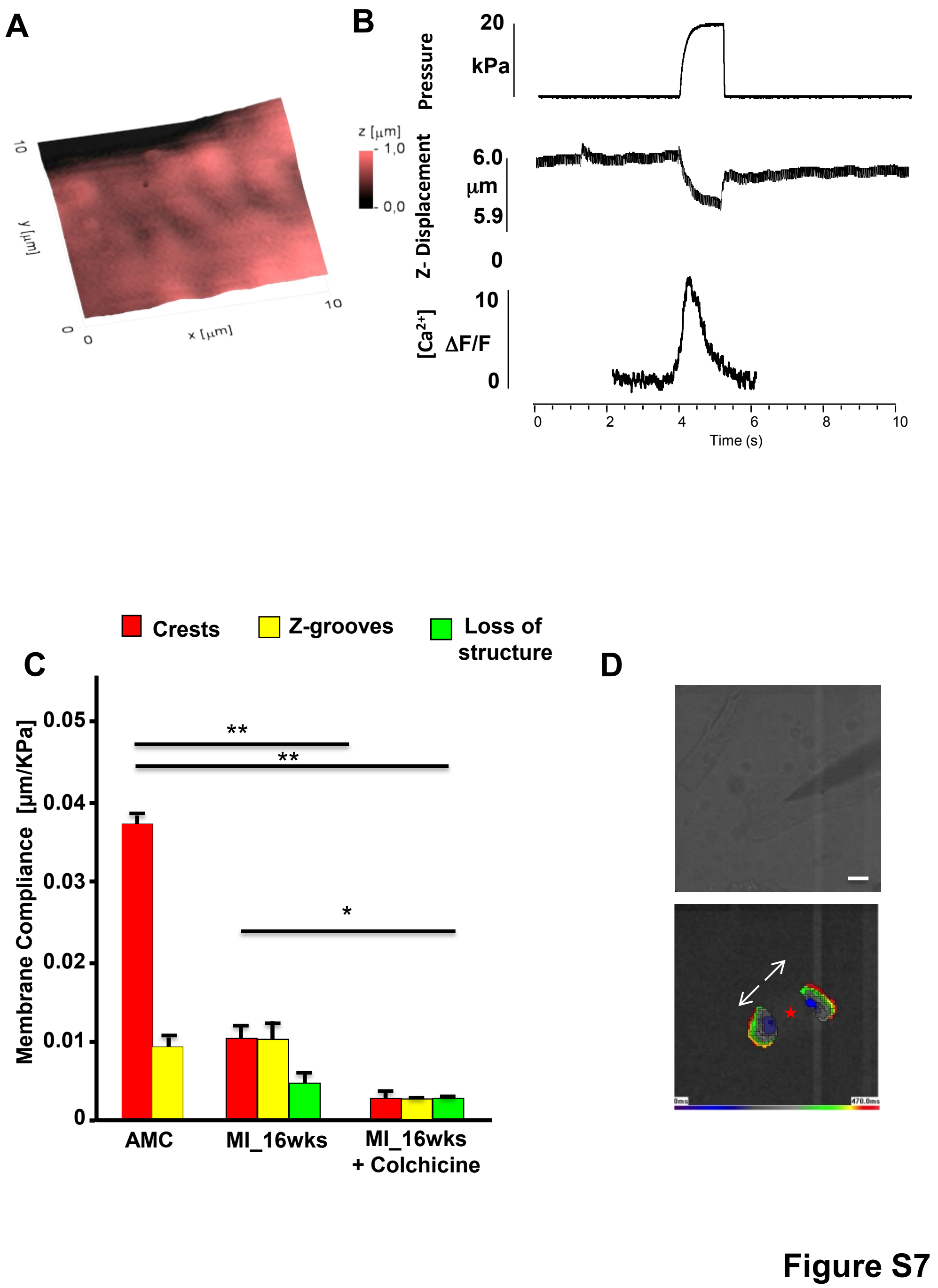

Figure S7, related to Figure 5

Effect of microtubular network derangement in failing cardiomyocyte. A. Representative SICM topographical image of a HF cell after colchicine treatment; **B**. Traces of mechanical application (top) membrane indentation (middle) and MiCa$_i$ transient (bottom) in failing cardiomyocytes subjected to colchicine. **C**. Membrane compliance measurement in control cardiomyocytes (AMC), failing cardiomyocytes and failing cardiomyocytes subjected to colchicine. **D**. Top: nanopipette positioned on top of a caridoymocyte for pressure application and (bottom) isochronic color-coded map of a subsequent MiCa$_i$ event.

Weeks Post MI	n	HW (mg) / TL (mm) [mean (SEM)]	n	LVEF (%)
Control	10	36.4 (2.07)	9	78.5 (3.21)
4	16	41.0 (1.10) *	5	53.5 (6.96)**
8	9	40.1 (1.43)	8	36.76 (5.90)***
16	16	46.5 (1.89) **	9	29.4 (2.02)***

Table S1

Table S1, related to Figure 2.

Validation of the heart failure model by generating myocardial infarction with coronary artery occlusion.

Heart weight/Tibia length ratio (HW/TL) and left ventricular ejection fraction (LVEF) in rats measured at 4, 8 and 16 weeks after coronary ligation. ** $P<0.05$ and *** $P<0.01$

Rat Cell Type	$MiCa_i$	Surface structure	Membrane Compliance in Crest / Groove / Unstriated regions (µm/kPa)	Z-Groove Ratio, % change from AMC cells
**AMC**	Focal at Z-Grooves	Regular	0.037 / 0.009 / -	100
**AMC + Colchicine**	Focal at Z-Grooves<br>Propagated at crests	Regular	0.01. / 0.005 / -	Unchanged
**AMC + Colchicine + CCCP**	None	Regular	0.014 / 0.006 / -	Unchanged
**MI-4 wks**	Focal at Z-Grooves;<br>Propagated with single initiation	Loss of some structures	0.009 / 0.006 / 0.003	-6.8
**MI-8 wks**	Propagated with triple initiation	Non-striated	0.008 / 0.015 / 0.010	-24.7
**MI-16 wks**	Propagated with triple initiation	Non-striated	0.010 / 0.010 / 0.013	-29.8
**MI-16 wks + Colchicine**	Propagated with triple initiation	Non-striated	0.003 / 0.002 / 0.003	-28.8
**MI-16 wks + Gd^{3+}**	Propagated with single initiation	Non-striated	0.015 / 0.010 / 0.011	-27.9
**MI-16 wks + CCCP**	none	Non-striated	0.008 / 0.006 / 0.001	-30.3

Table S2, related to Figure 3.**Summary of different conditions where we observed $MiCa_i$**

Supplemental Movie Legend

Movie S1. Pattern of $MiCa_i$ propagation in MI _16wks cardiomyocytes: single initiation

Movie S2. Pattern of $MiCa_i$ propagation in MI _16wks cardiomyocytes: multiple initiations.

Movie S3. Inhibition of mechanosensitive ion channels allows only single initiation of $MiCa_i$.

Supplemental Experimental Procedure

In-vivo cardiac function

Cardiac function was assessed via biometrics and echocardiography. Heart weight corrected to tibia length provided a measure of hypertrophy. Echocardiography was performed under general anaesthesia (2% isoflurane) immediately prior despatch to give a measure of in-vivo cardiac function. The imaging was performed in M-Mode in the parasternal long axis view (Table S1, Vevo 770 system). After 4, 8 or 16 weeks following coronary ligation, rats were despatched by cervical dislocation after brief exposure to 5% isoflurane until the righting reflex was lost. We perfused the left ventricle (LV) via the Langendorff perfusion apparatus[1]. Cardiomyocytes were enzymatically isolated from the LV.

Topographical images

High-resolution cell membrane topography was achieved using high-resistance nanopipette (100MΩ). Surface topographical images (10x10 μm, 512x512 pixels) of the cardiomyocytes were acquired by the SICM at 25°C, pH=7.4. In order to avoid contraction and the same preparation was superfused with HBSS, pH=7.4 at 36 °C afterwards. After acquiring the topography image, the pipette was moved to a selected location on the cell surface 200 nm above a cell crest or groove by a controlled movement of the piezo drive.

Transmission electron microscopy

Isolated cells were fixed in 2.5% glutaraldehyde in cacodylate buffer, cell pellets were embedded in 2% agarose and re-fixed in 2.5% glutaraldehyde, post-fixed in 1% osmium trioxide and embedded in Araldite following standard protocol. Ultra-thin sections were mounted on grids and stained for 7 minutes with 2% uranyl acetate in methanol, washed again in methanol, and stained for 5 minutes with 1% led citrate in water and washed in water afterwards. The sections were observed with transmission electron microscope.

Immunostaining

Adult cardiomyocytes were stained for β-tubulin (Monoclonal Anti-β -Tubulin; t5201; Sigma-Aldrich; Alexa 546 anti-mouse immunoglobulin; Abcam) by a standard indirect immunofluorescence protocol. Cells on coverslips were fixed in 4% paraformaldehyde in PBS for 10 minutes, permeabilized in 1% triton X100 in PBS for 20 minutes; washed in PBS twice, incubated in blocking solution (5% BSA, 20% Newborn calf serum, 0.05% Tween 20 in PBS) for 30 minutes They were then incubated with first antibody (diluted 1:100) in blocking solution overnight, washed in PBS twice, incubated with secondary antibody (diluted 1:100) in blocking solution for 30 minutes. They were subsequently mounted in Vectashield mounting medium (Vector Labs), sealed with nail varnish, and observed with confocal microscope (Zeiss LSM-780).

Supplemental reference.

1. Sato M, O'Gara P, Harding SE, Fuller SJ. Enhancement of adenoviral gene transfer to adult rat cardiomyocytes in vivo by immobilization and ultrasound treatment of the heart. *Gene Ther*. 2005;12:936-941
